# Selective gene expression analysis of the neuroepithelial body microenvironment in postnatal lungs with special interest for potential stem cell characteristics

**DOI:** 10.1186/s12931-017-0571-4

**Published:** 2017-05-08

**Authors:** Line Verckist, Robrecht Lembrechts, Sofie Thys, Isabel Pintelon, Jean-Pierre Timmermans, Inge Brouns, Dirk Adriaensen

**Affiliations:** Laboratory of Cell Biology and Histology, Department of Veterinary Sciences, University of Antwerp, Universiteitsplein 1, BE-2610 Antwerpen, Wilrijk Belgium

**Keywords:** Airway epithelium, Neuroepithelial body microenvironment, Pulmonary neuroendocrine cells, Stem cell niche, Laser microdissection, PCR array, Delta-like ligand 3

## Abstract

**Background:**

The pulmonary neuroepithelial body (NEB) microenvironment (ME) consists of innervated cell clusters that occur sparsely distributed in the airway epithelium, an organization that has so far hampered reliable selective gene expression analysis. Although the NEB ME has been suggested to be important for airway epithelial repair after ablation, little is known about their potential stem cell characteristics in healthy postnatal lungs. Here we report on a large-scale selective gene expression analysis of the NEB ME.

**Methods:**

A GAD67-GFP mouse model was used that harbors GFP-fluorescent NEBs, allowing quick selection and pooling by laser microdissection (LMD) without further treatment. A panel of stem cell-related PCR arrays was used to selectively compare mRNA expression in the NEB ME to control airway epithelium (CAE). For genes that showed a higher expression in the NEB ME, a ranking was made based on the relative expression level. Single qPCR and immunohistochemistry were used to validate and quantify the PCR array data.

**Results:**

Careful optimization of all protocols appeared to be essential to finally obtain high-quality RNA from pooled LMD samples of NEB ME. About 30% of the more than 600 analyzed genes showed an at least two-fold higher expression compared to CAE. The gene that showed the highest relative expression in the NEB ME, Delta-like ligand 3 (Dll3), was investigated in more detail. Selective Dll3 gene expression in the NEB ME could be quantified via single qPCR experiments, and Dll3 protein expression could be localized specifically to NEB cell surface membranes.

**Conclusions:**

This study emphasized the importance of good protocols and RNA quality controls because of the, often neglected, fast RNA degradation in postnatal lung samples. It was shown that sufficient amounts of high-quality RNA for reliable complex gene expression analysis can be obtained from pooled LMD-collected NEB ME samples of postnatal lungs. Dll3 expression, which has also been reported to be important in high-grade pulmonary tumor-initiating cells, was used as a proof-of-concept to confirm that the described methodology represents a promising tool for further unraveling the molecular basis of NEB ME physiology in general, and its postnatal stem cell capacities in particular.

**Electronic supplementary material:**

The online version of this article (doi:10.1186/s12931-017-0571-4) contains supplementary material, which is available to authorized users.

## Background

Neuroepithelial bodies (NEBs) occur in the airway epithelium as densely innervated clusters of pulmonary neuroendocrine cells (PNECs, for review see [1]). In most mammalian species (including humans) PNECs are crowned by Clara-like cells (CLCs), leaving only thin apical processes of PNECs in contact with the airway lumen [2, 3]. CLCs, PNECs and their extensive innervation together constitute the so-called NEB microenvironment (ME) [4–6].

Functional hypotheses for the NEB ME have originally been based on mainly morphological findings. The main prerequisite to perform large-scale functional morphological investigations on NEBs is their clear identification and visualization in the microscope. Today, immunofluorescent labeling of PNECs, combined with visualization by fluorescence and confocal microscopy, is a valuable tool to study the overall features of pulmonary NEBs (for review see [7]). Although different antibody markers are able to unambiguously identify NEBs, the recent application of a glutamic acid decarboxylase (GAD67)-GFP mouse model allows fast and unequivocal detection of GFP-fluorescent NEBs, even in live lung preparations, without major tissue manipulation [8].

Historically, the direct functional and molecular investigation of NEBs has been problematic. Because of their relatively low number (2000–3000 in a pair of mouse lungs) [9] and widespread distribution in the epithelium of intrapulmonary airways only, NEBs are virtually unreachable for direct measurements and manipulation. Whereas for functional studies, live cell imaging of the mouse NEB ME has been accomplished using ex vivo lung slice models [6, 10–14], reliable gene expression studies of the NEB ME are still sparse.

In the past, many efforts have been made to establish pure populations of PNECs using primary cultures or immortalised small cell lung carcinoma cell (SCLC) lines, which may however significantly differ from their counterparts in the natural environment [15–20]. A method that is designed to selectively and precisely dissect tissue regions or cell groups from tissue sections/freshly isolated organ preparations, seems to be a better option to obtain accurate molecular information from NEBs. The first aim of this study therefore was to optimize laser microdissection (LMD) for the selective isolation of NEBs and for consecutive complex gene expression analysis. Evidently, the ‘purity’ of the NEB sample, will need to be validated via the expression of marker proteins in mouse airways, i.e., calcitonin gene-related peptide (CGRP) [21, 22] and GAD67 [8] for NEB cells, Clara cell secretory protein (CCSP) for Clara cells and to a lesser extent also CLCs [4, 23, 24] and FMS-like tyrosine kinase (Flt-1) for ciliated cells [25].

As well-established for other cells and systems, it is hypothesised that the unique origin, structure and function of the NEB ME will be related to the distinct expression of genes, as compared to the surrounding control airway epithelium (CAE). Therefore, methods for mRNA expression analysis such as reverse transciption polymerase chain reaction (RT-PCR) or quantitative (real-time) RT-PCR (q(RT)-PCR; qPCR) and PCR arrays could provide valuable information to unravel the unique functional characteristics of the NEB ME.

Over the years, NEBs have been proposed to serve several functions in the regulation of physiological processes in the lungs during fetal, perinatal, and postnatal life [5, 7, 26–31]. In pre- and perinatal rabbits and mice extensive evidence implicates NEBs in initiating hypoxia-sensing and -transduction [10, 32, 33]. In postnatal mice there is clear evidence that NEBs act as a sensor, both for mechanical changes in the airway wall [34–36] and for local changes in extracellular compounds (such as Ca^2+^ or H^+^) [14, 37].

The NEB ME has been mentioned as one of the potential locations of stem cells that are dispersed along the respiratory tract [38–40]. NEBs have been proposed as a niche for airway epithelial stem/progenitor cells, responsible for the homeostasis of bronchiolar epithelium during normal turnover and repair of severe injury [38, 39, 41, 42]. In this respect, at least two distinct cell types should be distinguished in NEBs, i.e., CCSP^+^/CyP450^−^ CLCs and PNECs [4, 5, 12]. Although different studies have suggested an important role for NEBs in the changes seen during severe airway injury [43, 44] and in lung disease, e.g., SCLC [45–47], little is known about the potential postnatal stem cell characteristics of the NEB ME.

In view of further unraveling unique features of the NEB ME as a potential stem cell niche in healthy postnatal mouse lungs, the second aim of the present study was large-scale selective gene expression analysis of NEBs using several commercially available PCR arrays. The arrays were selected based on known involvement of the genes in development, stem cell behavior and signaling, allowing access to a panel of more than 600 genes. Aiming at a thorough comparison between the NEB ME and intrapulmonary CAE, each PCR array was performed for both a pooled NEB ME sample and a CAE sample collected from the same mouse lung by LMD. For LMD, cryosections of the lungs of GAD67-GFP mice [8] were used, allowing fast and reliable selection of NEBs. For genes that showed a higher expression in the NEB ME compared to CAE, a top 20 was made based on the relative expression level. To obtain more detailed information about individual genes of interest from the PCR arrays, custom primers need to be developed for validation of the gene expression analysis using qPCR experiments with RNA samples of multiple animals, and quantification based on the expression of an optimal set of reference genes. As a proof-of-concept, we include a detailed analysis, including protein localization, of the gene with the highest relative expression in the top 20.

## Methods

### Animals

Lung and brain tissue were obtained from wildtype C57-Bl6 (*n* = 5) and GAD67-GFP C57-Bl6 mice (*n* = 20) [8] (The Jackson Laboratory, Charles River, Saint-Germain-sur-l’Arbresle, France) on postnatal day (PD) 21, and lung tissue on embryonic day (ED) 14 (*n* = 2), PD0 (= day of birth; *n* = 2), and from 3-months-old (= adult; *n* = 2) animals. All animals were housed with their mothers in acrylic cages in an acclimatized room (12/12 h light-dark cycle; 22 ± 3 _˚_C) and were provided with water and food ad libitum. National and international principles of laboratory animal care were followed, and experiments were approved by the local animal ethics committee of the University of Antwerp (2010–38, 2011–48, 2014–66). All animals were killed by intraperitoneal injection of an overdose of sodium pentobarbital (Nembutal 200 mg/kg, CEVA Sante Animale, Brussels, Belgium).

### Laser microdissection (LMD)

Lungs of GAD67-GFP mice were intratracheally filled with a general RNAse inhibitor (SuperaseIn®, 400 U/ml, Fisher Scientific, Aalst, Belgium) containing 0,1% paraformaldehyde, dissected, immediately snapfrozen in liquid nitrogen and stored at -80 °C. Five 20-μm-thick cryosections are thaw-mounted on each polyethylene terephthalate (PET) Frameslide (Leica, Wetzlar, Germany), immediately refrozen and kept on dry ice until further use. The lungs of GAD67-GFP mice harbor GFP-fluorescent NEBs [8] that can be visualized using an appropriate fluorescence filter combination (Leica LMD-GFP band pass/Leica LMD-GFP long pass). Slides were dehydrated in ethanol prior to LMD.

**Fig. 1:**
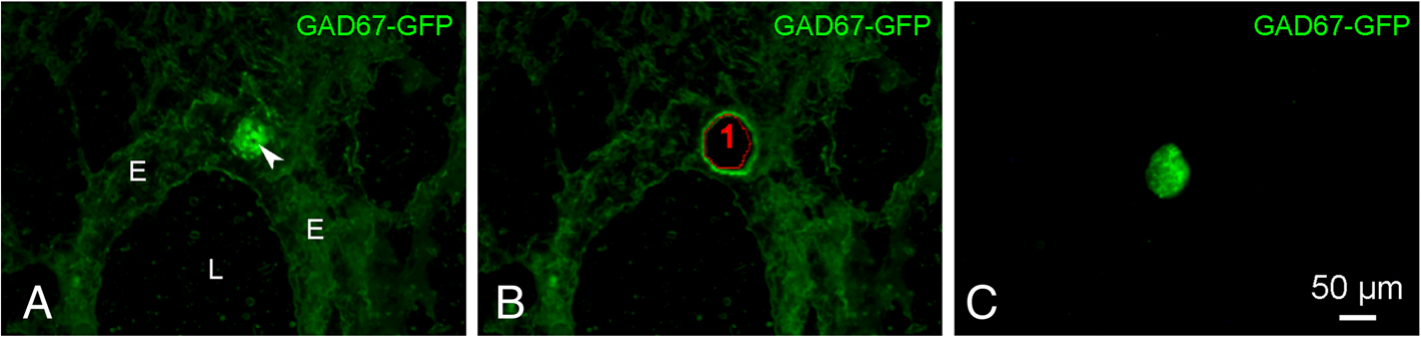
Example of the isolation of a pulmonary NEB from a GAD67-GFP mouse lung cryosection using LMD (see Methods). **a** GFP-fluorescent NEB (*arrowhead*) located in the epithelium of an intrapulmonary airway. **b** Image taken after LMD. The area circled by the *red* line shows the region of interest that was selected to be cut by the laser. **c** Isolated GFP-fluorescent NEB, captured in the cap of an Eppendorf tube and ready for consecutive pooling and RNA isolation. Note that even after very mild fixation, to optimally preserve RNA quality, and without cover glass, NEBs appear to be unambiguously detectable in the LMD microscope (Leica LMD7000; 20x objective). *L: airway lumen, E: airway epithelium*

In short, the LMD system (Leica LMD7000) is based on a fully automated upright research microscope, thereby uniquely using the high-precision microscope optics to steer the UV-laser excision beam along the desired cut line without moving the slide. The design allows collection of the laser microdissected specimens purely based on gravity, and hence contamination free. In our case, the cut lung specimens will drop and are captured/pooled into the cap of a 0.2 ml Eppendorf tube that is automatically positioned under the PET Frameslide (also see Fig. 1). Since the cap is prefilled with RTL Plus lysis buffer and β-mercaptoethanol (Qiagen, Hilden, Germany), no additional manipulation is required to further process the specimens for RNA isolation.

Depending on the number of GFP-fluorescent NEBs in the section, 2–5 samples of NEB ME and one sample of CAE are collected from every cryostat section. These are respectively pooled to a total of 300 NEB ME samples and 25 CAE samples. Additionally, two whole cryostat sections were taken as a control for the impact of the LMD technique on the quality of consecutively isolated RNA. RNA isolation is performed on independently pooled samples of the NEB ME and CAE and on sections of whole lung tissue, collected from the same mouse. All procedures are repeated for several mice.

### RNA isolation

The pooled LMD samples were used to isolate total RNA (RNeasy Plus Micro kit; Qiagen). Concentration and integrity of every RNA sample was examined by an Agilent 2100 Bioanalyzer electrophoresis system (Agilent Technologies, Waldbronn, Germany), after preparation with an Agilent RNA 6000 Pico Kit. Data were analyzed using gel-like images (bands, compared to fragments of a known ladder) and electropherograms. Additionally, an internal standard is added to the run to calibrate the ladder data and sample data. The RNA integrity number (RIN) is calculated, which gives an indication of the over-all integrity of the RNA sample.

### Reverse transcription and amplification

Reverse transcription and amplification was performed on pooled NEB ME and CAE samples, and on samples of whole lung tissue by the Ovation PicoSL WTA System V2 kit (NuGEN Technologies, Leiden, The Netherlands) on an MJ Mini Cycler (Bio-Rad, Temse, Belgium) starting from picograms of total RNA. Amplification can start randomly throughout the whole transcriptome in the sample, making the protocol ideal for amplification of short strands of RNA. First, single stranded cDNA is prepared, resulting in a cDNA/mRNA hybrid molecule with a unique RNA tag sequence (SPIA tag) at the 5 ´ end of the cDNA strand. Next, fragmentation of the mRNA within the cDNA/mRNA complex initiates synthesis of a second cDNA strand after adding DNA polymerases, and creates a double-stranded cDNA with a DNA/RNA heteroduplex corresponding to the SPIA tag at one end. Adding DNA polymerases starts the synthesis of cDNA at the 3′ end of the primer, removing the existing forward strand. Repetition of this process, by using a defined program of consecutive thermal steps for the SPIA DNA/RNA primer binding, DNA replication, strand displacement and RNA cleavage, results in a rapid amplification of cDNA.

### Quantitative (real-time) RT-PCR

SYBR Green expression analysis was used for qPCR analysis in a multiwell-plate-based system (LightCycler480; LC480; Roche Applied Science, Penzberg, Germany).

Primer design was based on the general guidelines for real-time PCR primer design following the Minimum Information for Publication of Quantitative Real-Time PCR Experiments (MIQE) guidelines [48]. A BLAST analysis was performed to confirm the specificity of the primers. Almost all primers were designed to be intron-spanning to prevent the replication of residual contaminating DNA, and to obtain an amplicon length of 60–150 bp.

Real-time PCR was performed on 5 μl cDNA using the LightCycler® 480 SYBR Green I Master (Roche Applied Science) in a final reaction volume of 20 μl in LC480 white 96 Multiwell Plates (Roche Applied Science). All samples were run in triplicate and included a no-template control. The efficiency of each developed primer set is initially confirmed with a standard curve to exclude possible DNA contamination and/or PCR inhibition, problems with the sample quality, or pipetting problems. This efficiency is determined by the slope of a standard curve based on a serial dilution (5-point 4-fold dilution) of cDNA.

Reactions were carried out as follows: after an initial preincubation of 5 s at 95 °C, amplifications consisted of 40 cycles of denaturation at 95 °C for 10 s, annealing of the primers at 60 °C for 15 s and elongation at 72 °C for 6 s. After obtaining the melting curves, the whole cycle was ended with a cooling step at 40 °C for 10 s.

The purity of LMD-obtained samples was characterized via expression analysis of selective marker genes for the collected pulmonary cell types, i.e., CGRP and GAD for NEB cells, CCSP for Clara cells and CLCs, and Flt-1 for ciliated cells. The eukaryotic translation elongation factor 2 (eEF2), ribosomal protein L38 (Rpl38) and L4 (Rpl4) were selected as reference genes from a series of candidate reference genes based on literature data [49], and use of the geNorm analysis implemented in qbase + (Biogazelle, Ghent, Belgium). These reference genes were used for normalization with the NEB ME sample as calibrator group [50]. Relative gene expression differences (qPCR) between pooled LMD samples of NEB ME and of CAE (GAD67-GFP mice; PD21), were calculated using the ΔΔCT-method [51]. Relative gene expressions are reported as the mean value ± SD. Amplification products were separated on a 2% agarose gel and visualized under UV illumination. Custom primer sequences are listed in Table 1.

**Table 1:** List of primers (FP = forward primer, RP = reverse primer) used for qPCR. *Dll3: Delta-like ligand 3*

Gene	Primer/Probe	Sequence (5’ → 3’)
eEF2	FP	TAAGGAGGGCGCTCTCTGTGAGG
	RP	TGGCCACCTCCCCGGTGAAT
Rpl38	FP	ACAGACAAGGAAAAGGCAGAG
	RP	TTTAATAGTCACACGCAGAGGG
Rpl4	FP	AACACCGACCTTAGCAGAATC
	RP	ATAGTCTTGGCGTAAGGGTTC
CCSP	FP	GCTGCAGCTCAGCTTCTTCGGA
	RP	GGTCTGAGCCAGGGTTGAAAGGC
CGRP	FP	GGAGGCTGAGGGCTCTAGTGTC
	RP	CAAAGTTGTCCTTCACCACACCTC
GAD	FP	CCATCCAACGATCTCTCTCATC
	RP	ACATCGACTGCCAATACCAATA
Flt-1	FP	GCACGGGAGAGACTGAAACTAGG
	RP	GATCTTGAGTTCGGTCATCAGAGCT
Dll3	FP	AGGTTACAAGACGGTGCTGG
	RP	GATCAGGCCTCTCGTGCATA

### PCR Arrays

For a more comprehensive study of the potential stem cell characteristics of the postnatal NEB ME, different commercially available PCR arrays that are related to stem cell behavior and development were used. The selected RT^2^ Profiler™ PCR Arrays (Mouse; Qiagen, Les Ulis, France) are shown in Table 2, and for each array all included genes are listed in Additional file 1: Table S1.

**Table 2:** Selected RT^2^ Profiler™ PCR Arrays (Qiagen), based on the inclusion of primers for studying the expression of genes that are potentially involved in stem cell behavior and development in mice. Each array experiment was simultaneously carried out for a NEB ME sample and a CAE sample, collected from the same mouse lung via LMD

RT^2^ Profiler^TM^ PCR array	Catalog number
Cancer stem cells	PAMM-176Z
Growth factors	PAMM-041Z
Hedgehog pathway	PAMM-078Z
Hippo signaling pathway	PAMM-172Z
Notch signaling pathway	PAMM-059Z
Signal transduction pathway finder	PAMM-014Z
Stem Cell	PAMM-405Z
Stem cell signaling	PAMM-047Z
TGFβ/BMP pathway	PAMM-035Z
TGFβ signaling targets	PAMM-235Z
Wnt signaling pathway	PAMM-043Z

The RT^2^ Nano PreAMP cDNA Synthesis Kit (Qiagen) is used for the multiplex PCR-based preamplification of the selectively pooled RNA samples of NEB ME and CAE. In consecutive steps, genomic DNA is eliminated and a first cDNA strand is synthetized from the initial RNA, this single strand is amplified using a PCR-based amplification step, and a side reaction reducer is added to inhibit residual primers after pre-amplification.

The 96-well array plate contains 84 array-specific primer sets for the genes of interest and additional controls, including reference genes for data normalization, and detectors for genomic DNA contamination, RNA sample quality and general performance of the PCR experiment. A first 10 min step at 95 °C is required to activate the hot-start iTaq DNA polymerase and is followed by amplifications that consist of 45 cycles of denaturation at 95 °C for 15 s, and annealing/elongation of the primers at 60 °C for one minute. Relative gene expression differences were calculated using the ΔΔCT-method and RT^2^ Profiler™ PCR Array Data Analysis 3.4 software (Qiagen). A threshold for the fold regulation of more than 4 was chosen as a cut-off value to define a ‘higher expression’ of genes in the NEB ME, as compared to CAE. Although it will not be considered in the present proof-of-concept study, we evidently realize that also genes with a clear down-regulation in the NEB ME, compared to CAE, will be of interest.

### Immunohistochemical staining of lung cryosections

For immunohistochemical staining, lungs of GAD67-GFP and wildtype C57-Bl6 mice were transcardially perfused with standard physiological solution and subsequently filled with 4% paraformaldehyde (PF) via the trachea. Lungs, trachea, esophagus and heart were dissected *en bloc*, deaerated in a mild vacuum, and immersion-fixed in the same fixative for 30 min. After rinsing in phosphate-buffered saline (PBS; 0.01 M; pH 7.4), tissues were stored overnight in 20% sucrose (in PBS; 4 °C), and mounted in Tissue-Tek O.C.T. (#4583, Sakura Finetek Europe, Zoeterwoude, The Netherlands). Cryostat sections (20 μm thick) of the whole tissue blocks were thaw-mounted on poly-L-lysine-coated microscope slides, dried at 37 °C (2 h) and processed for immunolabeling. Immunohistochemical incubations were performed in a closed humidified container (room temperature). All primary and secondary antisera were diluted in PBS containing 10% normal horse serum and 0.1% BSA (PBS*). Before incubation with the primary antiserum, cryostat sections were permeabilized for 1 h with PBS* containing 1% Triton X-100. Sections were then incubated overnight with the primary antibodies (Delta-like ligand 3 (Dll3); rabbit polyclonal; Novus, Missouri, USA; dilution 1/100). For visualization of the immunostaining, sections were rinsed and further incubated for 4 h with the secondary antibodies (Cy™3 -conjugated Fab fragments of goat anti-rabbit IgG; 111–167-003, Jackson ImmunoResearch, Suffolk, UK; dilution 1/2000), and after a final wash in PBS mounted in Citifluor (Ted Pella, Redding, CA, USA). Negative staining controls for all immunocytochemical procedures were performed, by substituting the primary antisera with non-immune sera.

### Microscopic data acquisition and analysis

An epifluorescence microscope (Zeiss Axiophot, Carl Zeiss, Jena, Germany) equipped with filter cubes for the visualization of FITC/GFP (Zeiss 17; BP475-495/FT510/BP515-565) and Cy3 (Zeiss 14; LP510-KP560/FT580/LP590) was used to quickly screen the immunostaining results. All high-resolution images were obtained using a microlens-enhanced dual spinning disk confocal microscope (Ultra*VIEW* VoX; PerkinElmer, Zaventem, Belgium) equipped with 488 nm and 561 nm diode lasers for excitation of FITC/GFP and Cy3. Images were acquired and processed using Volocity 6.3.1 software (PerkinElmer).

## Results

### Laser microdissection for obtaining selective samples of the NEB ME

To allow easy and fast identification of pulmonary NEBs from other areas of airway epithelial cells, lungs of GAD67-GFP mice, which in the airways selectively express GFP in PNECs, are used. Intrapulmonary fixation by instillation of 0,1% PF (5 min) via the trachea, allows the straightforward visualization of GFP-fluorescent NEBs in non-coverslipped cryostat sections on PET Frameslides (Fig. 1). Due to some background fluorescence, an adequate identification of CAE is also allowed. Combined with LMD, this protocol was shown to permit a selective collection of samples of the NEB ME, with a minimum of ten NEBs per frame slide. The RNeasy Plus Micro kit is especially developed for purification of total RNA from small samples (≤5 × 10^5^cells) that are microdissected. Nevertheless, purification of RNA from less than a 100 cells can lead to stochastic problems with respect to copy number. Therefore, pooling of samples of the NEB ME was performed to obtain about 300 NEBs as starting material for RNA purification. Similarly, around 25 pieces of CAE are collected via LMD and pooled in 350 μl lysis buffer. RNA isolation from the pooled samples collected via LMD results in an mRNA yield of 300–800 pg/μl for the NEB ME samples (3.6–12 ng total RNA) and 500–900 pg/μl for CAE samples.

**Fig. 2:**
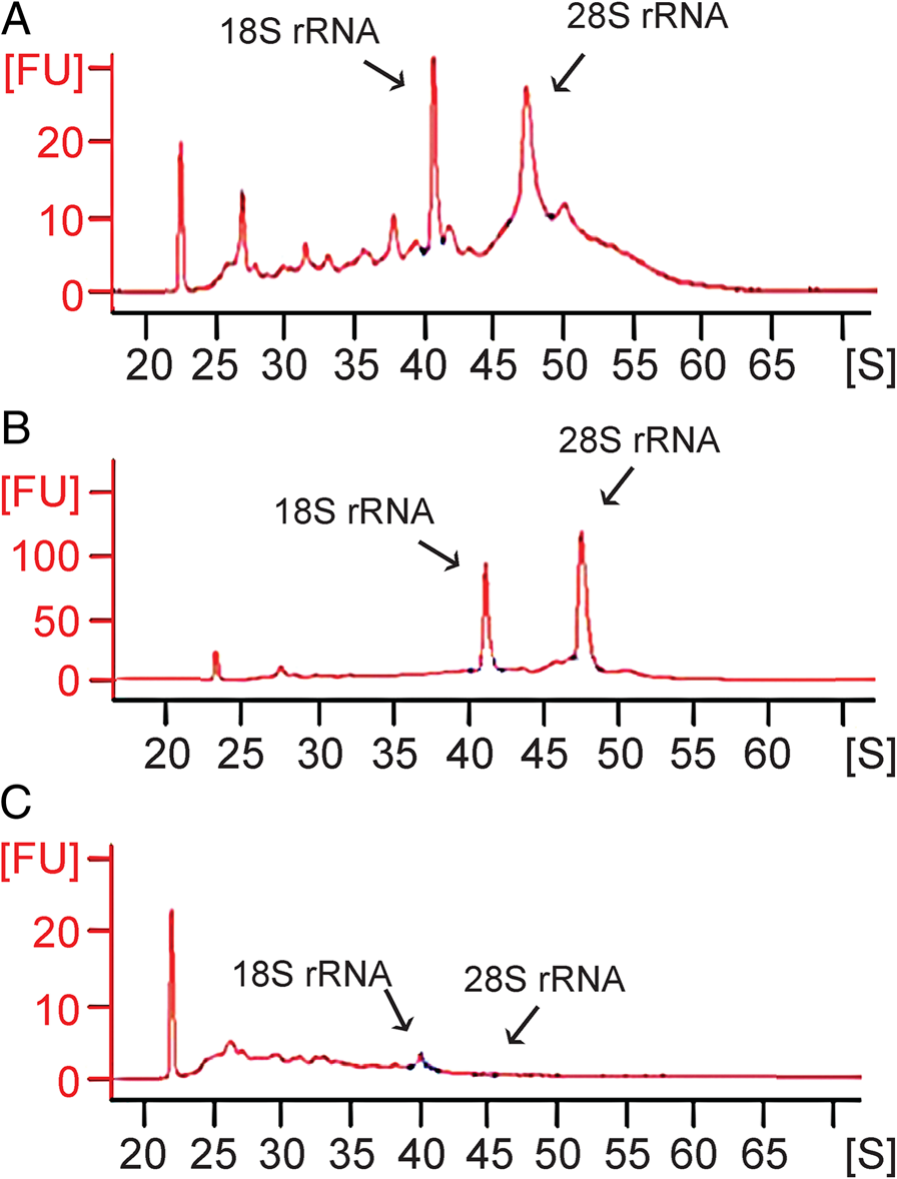
Electropherograms demonstrating the 18S and 28S rRNA peaks, corresponding to the level of intact RNA in each sample, are used for total RNA quality analysis of random LMD-collected and pooled small samples from cryostat sections of brain (PD21; **a**), embryonic (ED14; **b**) and postnatal lung (PD21, **c**). In the brain (RNA Quality Indicator, RIN = 7.9) and embryonic mouse lung (RIN = 8.9), high quality intact RNA can be detected, while in the identically processed postnatal mouse lung tissue a large part of the RNA appears to be degraded (RIN = 3.2)

Preliminary RNA integrity studies (Fig. 2) showed that pooled small LMD samples of cryosections of brain (RIN = 7.9) and embryonic lung tissue (RIN = 8.9) yield mostly intact RNA, while in postnatal lungs RNA appeared to be highly degraded (RIN = 3.2).

**Fig. 3:**
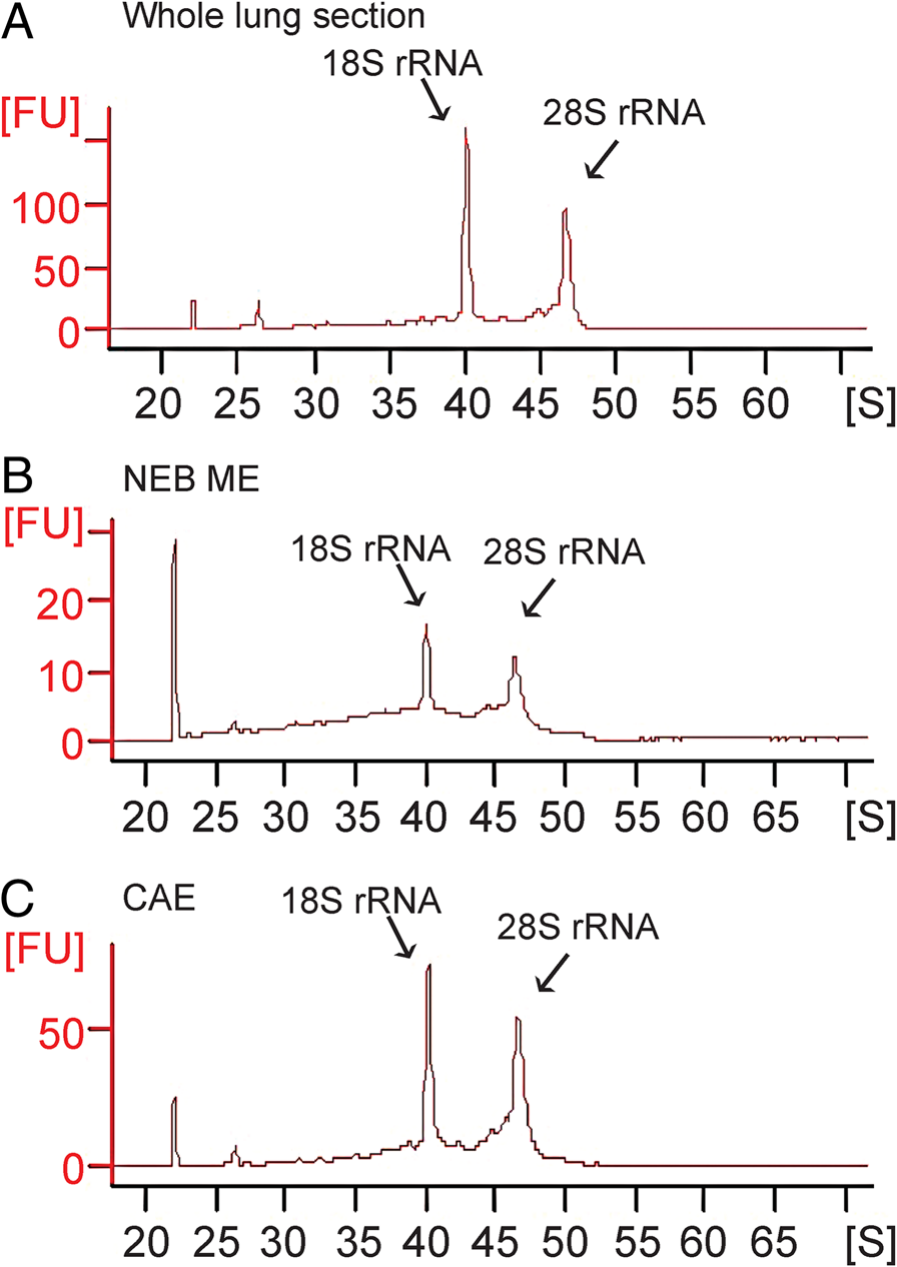
Electropherograms of a postnatal whole lung LMD section (**a**), pooled NEB ME (**b**) and CAE (**c**) samples that were obtained after optimized tissue processing, LMD and RNA isolation protocols. For all samples, the concentration of RNA was calculated by determining the area under the entire RNA electropherogram

Addition of an RNAse inhibitor (SuperaseIn®) to the fixative, and maximal reduction of the duration of aqueous phases in the protocols, however, appeared to result in a considerable increase of the amount of intact RNA (RIN values typically higher than 7) that could be retained in postnatal whole lung samples (Fig. 3). Compared to whole lung, NEB ME and CAE samples both show a slightly lower RIN value, which may be ascribed to the lower total concentration of RNA in these samples. For NEB ME samples RIN values typically vary between 5 and 7, while they are around 7 for CAE (Fig. 3). For all following experiments, only samples with sufficiently high RNA integrity (RIN value higher than 6.5) were further processed.

### Evaluation of the necessity of using LMD for analysis of genes with site-specific expression in the NEB ME

In this part of the study, we tried to confirm gene expression for two established marker proteins that in the lung are selectively localized in PNECs of the NEB ME, i.e., CGRP (an abundant neurotransmitter peptide) and GAD (low expression enzyme).

**Fig. 4:**
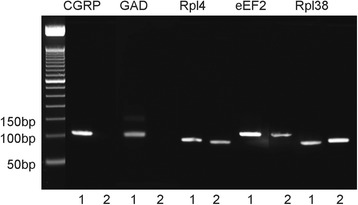
Non-amplified RNA samples. Detection of CGRP, GAD and reference genes’ expression in NEB ME (lane 1) and in a whole lung section (lane 2) of a GAD67-GFP mouse (PD21). The amplicon length of CGRP is 105 bp and that of GAD is 102 bp, both clearly visible as a distinct band on the gel electrophoresis image. For the whole lung sample, there is no visible band that can account for the CGRP or GAD amplicon, while there are positive bands for the NEB RNA sample. The reference genes are expressed in every sample

In non-amplified whole lung RNA samples, neither CGRP nor GAD expression could be detected, while expression of the reference genes was okay (Fig. 4). In contrast to whole lung samples, a strong CGRP and a weaker GAD expression could be shown in non-amplified RNA samples of LMD-selected and pooled NEB ME (Fig. 4), clearly pointing out the necessity of a more selective collection of samples for expression analysis of genes that are expressed only in rather rare cell groups with very specific locations.

**Fig. 5:**
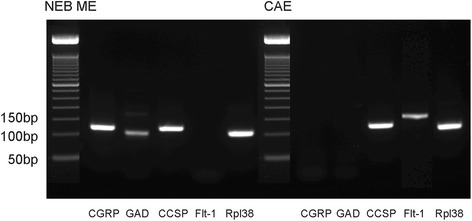
Gene expression of CGRP, GAD, CCSP, Flt-1 and Rpl38 in amplified samples of the NEB ME and CAE. CGRP and GAD are selective markers for neuroendocrine cells and gene expression can be seen in the NEB ME only. CCSP is a marker for both CLCs and Clara cells, and gene expression can be seen in NEB ME and CAE samples. Flt-1 is a selective marker for ciliated cells, which are present in the CAE sample only. Rpl38 is shown as reference gene and is strongly expressed in the NEB ME and in CAE

Additionally, a higher concentration of RNA is necessary to obtain enough starting product for a quantitative molecular analysis with sufficient internal controls. Hence pooled LMD samples were further subjected to an RNA amplification step, using the Ovation® WTA pico sytem V2 kit. After amplification, dscDNA (about 10 μl) could be measured with concentrations between 0.5 and 1 μg/μl, indicating a more than 100-fold amplification of the initial sample amount (see higher). In amplified NEB ME samples, no obvious changes were seen for CGRP and GAD expression (Fig. 5), while both NEB ME marker genes remained undetectable in amplified RNA samples of whole lung (not shown). The mRNA yield of the starting product was high enough to run multiple gene expression experiments in triplicate and with the optimized panel of reference genes. Moreover, amplification also allows large-scale gene expression profiling, which needs micrograms of total RNA, to be performed for NEB ME samples after the RNA amplification step.

### Assessment of quality and purity of LMD samples of CAE and NEB ME

To confirm whether the optimized LMD protocols allow selective isolation and pooling of either NEB ME samples or CAE samples from postnatal airway epithelium, qPCR was performed on amplified RNA samples for genes that are known to be selectively expressed in the NEB ME (i.e., PNECs and CLCs), and CAE (i.e., ciliated cells and Clara cells). Tested genes were CGRP (marker for mouse NEB cells; [21, 22, 52], Flt-1 (marker for mouse ciliated cells; [25]) and CCSP (marker for Clara cells, but also for CLCs [52]). Amplification products were separated on a 2% agarose gel and visualized under UV illumination to detect the specified amplicons.

Clear mRNA expression for CGRP and GAD can be detected in the NEB ME but not in CAE, while Flt-1 mRNA expression was present in samples of CAE but not in NEB ME samples (Fig. 5). CCSP mRNA is expressed both in CAE, due to the presence of Clara cells, and in NEB ME samples, due to the presence of CLCs. These data show that differential gene expression of CGRP, Flt-1 and CCSP is sufficient to evaluate the straightforward discrimination and isolation of pooled NEB ME and CAE samples from postnatal lung sections by our optimized LMD protocols.

These three marker genes are now routinely used to test for the selectivity/purity of all newly isolated and pooled NEB ME and CAE samples.

### Expression of stem cell-related genes in the NEB ME

To allow rapid identification of differential expression of stem cell-related genes in the NEB ME, expression levels were compared between amplified mRNA samples of the NEB ME and CAE using different commercially available PCR arrays (see Table 2). In this manner, a panel of more than 600 genes was constituted and selected based on their potential involvement in stem cell behavior or in pathways that are known to be implicated in lung development or repair. The raw expression data for all genes and arrays are listed in Additional file 1: Table S1.

A large number of stem cell-related genes showed a higher expression in the NEB ME compared to CAE. Of the analyzed genes, 181 (30,1%) showed an at least two-fold higher expression in the NEB ME compared to CAE. Even using a threshold of four, to obtain a more reliable fold regulation, still 13.7% of the analyzed genes fit in. Table 3 includes a top 20 of the genes that show the highest upregulation of gene expression in the NEB ME.

**Table 3:** Expression levels of the top 20 of potential stem cell-related genes that showed a higher mRNA expression in the NEB ME compared to CAE in PCR arrays

Gene	Gene name	Fold regulation	Location	Function	Family
Dll3	Delta-like ligand 3 (Drosophila)	359.86	Membrane	Developmental protein	Notch
Mapk10	Mitogen-activated protein kinase 10	257.01	Cytoplasm	Serine/threonine-protein transferase	Protein kinase
Lef1	Lymphoid enhancer binding factor 1	69.31	Nucleus	Transcription regulation	Wnt
Lor	Loricrin	58.95	Cytoplasm	Structural constituent of cytoskeleton	TCF/LEF
Fgf14	Fibroblast growth factor 14	40.80	Nucleus	Growth factor	Hepann-binding growth factor
Fgf5	Fibroblast growth factor 5	39.69	Secreted	Growth factor, mitogen	Heparin-binding growth factor
Disp2	Dispatched homolog 2 (Drosophila)	29.24	Membrane	Component of membrane	Dispatched
Bmp2	Bone morphogenetic protein 2	27.10	Secreted	Cytokine, developmental protein, growth factor	TGFß
Plg	Plasminogen	25.50	Secreted	Hydrolase, protease, serine protease	Peptidase S1
Wnt16	Wingless-related MMTV integration site 16	19.57	Cytoplasm	Developmental protein	Wnt
Spp1	Secreted phosphoprotein 1	17.28	Secreted	Cytokine	Osteopontin
Nanog	Nanog homeobox	13.64	Nucleus	Developmental protein, transcription regulation	Nanog homeobox
Mstn	Myostatn	13.09	Secreted	Cytokine, growth factor	TGFß
Ptgs2	Prostaglandin-endoperoxide synthase 2	12.75	Membrane	Oxidoreductase. peroxidase	Prostaglandin G/H synthase
ll10	Interleukin 10	11.73	Secreted	Cytokine	ll10
Gli3	GLI-Kruppel family member GLI3	11.27	Nucleus	Transcription regulation	GLI C2H2-type zinc-finger protein
Bmp7	Bone morphogenetic protein 7	11.21	Secreted	Cytokine, developmental protein. gro\Mh factor	TGFfß
Fgf22	Fibroblast growth factor 22	10.71	Secreted	Growth factor	Hepann-binding growth factor
Igf2	Insulin-like growth factor 2	9.19	Secreted	Growth factor, hormone, mitogen	Insulin
Fgf9	Fibroblast growth factor 9	8.28	Secreted	Developmental protein, growth factor, mitogen	Heparin-binding growth factor

The gene that showed the highest expression in the NEB ME compared to CAE was Dll3, a member of the Notch signaling pathway that was included in two of the performed arrays. Both arrays showed a more than 50-fold upregulation for the expression of Dll3 in the NEB ME samples.

Dll3 is a transmembranous Notch ligand and can manage progenitor pools. Furthermore, a number of other genes that are involved in the Notch pathway are seen to have a higher expression in the NEB ME than in CAE (Table 4).

**Table 4:** Selected results of the PCR array for the Notch pathway, the table gives the fold regulation of the genes with the strongest upregulated expression in the NEB ME, as compared to CAE

Gene	Gene Name	Fold regulation
DII3	Delta-like 3	359.86
Lor	Loricrin	58.95
Sel1l	Sel-1 suppressor of lin-12-like (C. elegans)	7.52
Pax5	Paired box gene 5	6.83
Runx1	Runt-related transcription factor 1	5.75
Notch2	Notch2	5.00
Mfng	Manic Fringe Homolog	4.32
Krtl	Keratin 1	3.98

Although the PCR array data certainly provide valuable indications for gene expression profiles, it is tricky to make interpretations about the expression of individual genes. It is therefore necessary to confirm and further analyze the expression of genes of interest from the arrays in a reproducible and quantitative way using single qPCR, with proper controls and reference genes.

### Detailed analysis of the expression and localization of Dll3 in the NEB ME as a proof-of-concept for the reported work flow

#### Gene expression of Dll3 in LMD samples of the NEB ME and CAE

Expression of the Dll3 gene in the NEB ME was repeatedly measured, and chosen as a proof-of-concept for the reliability of the reported optimized workflow, LMD protocol and consecutive gene expression studies.

**Fig. 6:**
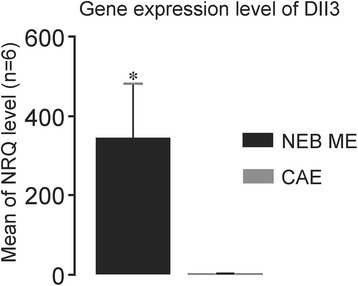
Results of qPCR experiments for the expression of Dll3. The mean expression in LMD samples of the NEB ME of five different mice is compared with matched CAE samples. The NRQ shows Dll3 mRNA levels in the NEB ME, as compared to the levels in the CAE, and normalized to expression of the reference genes Rpl38, eEF2 and Rpl4 in the same five samples. NRQ: Normalized Relative Quantification, n = 5. Differences are considered significant: p < 0,05 (*), using Mann-Whitney U test

A custom-designed primer set was used to analyze gene expression of Dll3. Dll3 mRNA levels in LMD samples of the NEB ME and CAE were quantified using single qPCR, and were normalized based on the expression level of different reference genes, i.e., Rpl38, eEF2 and Rpl4. The qPCR efficiency always showed values between 1.8 and 2.0. Dll3 expression appeared to be virtually absent in the intrapulmonary CAE, while relatively high levels of Dll3 mRNA could be measured in the NEB ME (Fig. 6).

#### Immunostaining for the Dll3 protein in mouse lungs

**Fig. 7:**
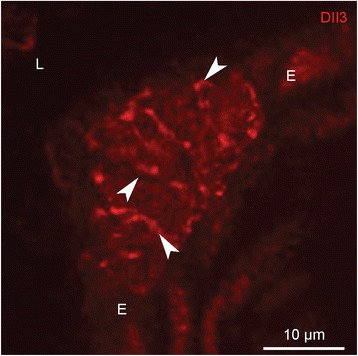
Single immunostaining for Dll3 (*red* Cy3 fluorescence) in a cryosection of the lungs of a wildtype mouse (PD21). Dll3 shows a spot-like staining (*arrowheads*) at distinct locations in the airway epithelium, covering the surface membrane of groups of intraepithelial cells. *L: airway lumen, E: airway epithelium*

To locate Dll3 protein in mouse lungs, immunohistochemical staining was performed. Dll3 immunoreactivity appeared to be limited to distinct areas within the airway epithelium (Fig. 7). More specifically, Dll3 antibodies could be shown to selectively stain the surface membrane of epithelial cells in a dotted pattern, a localization that may be expected for a transmembrane ligand (Fig. 7).

**Fig. 8:**
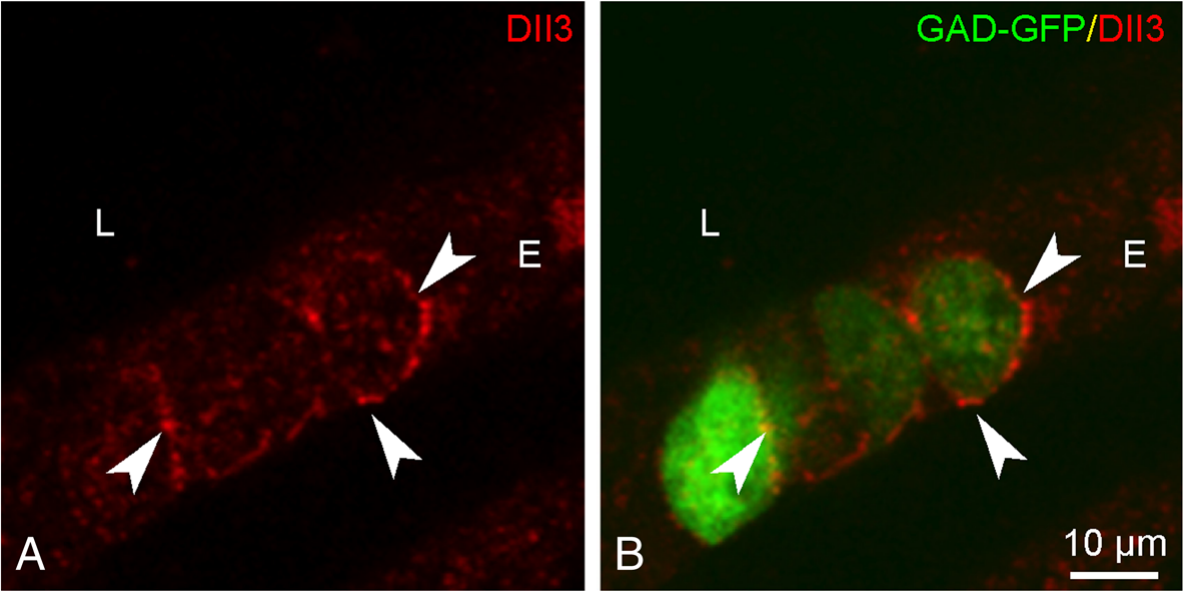
Dll3 immunostaining (*red* Cy3 fluorescence; **a**) on a lung cryosection of a GAD67-GFP mouse that shows GFP-expressing (*green* GFP fluorescence; **b**) NEB cells. Typically a spot-like Dll3 staining of the surface membrane of NEB cells can be observed (*arrowheads*). *L: airway lumen, E: airway epithelium*

Additionally, staining was performed on lungs of GAD67-GFP mice (PD21). Because free GFP fills the whole NEB cell [8], the Dll3 staining pattern on the surface membranes of the neuroendocrine cells is clearly visualized (Fig. 8). Dll3 protein expression was also found to be present in NEB cells of all other investigated postnatal ages (not shown).

## Discussion

The main interest of our research group concerns pulmonary NEBs, which are small cell groups that are widespread throughout the airway epithelium, and cannot be identified in light microscopes without specific labeling (mainly by immunohistochemistry). To obtain RNA from such specific subgroups of cells, laser microdissection (LMD) is a powerful tool, provided that quick and precise selection of the cell population is possible, and a good RNA integrity is maintained. Since preserving RNA integrity is challenging in postnatal lungs, an important part of the present study was focused on optimizing the complete workflow and LMD protocols for isolation and pooling of large numbers of small epithelial pieces from postnatal mouse lungs.

Good RNA integrity is essential for mRNA expression analysis using qPCR or microarrays [53]. RNA is, however, an unstable molecule that is rapidly degraded by very stable RNase enzymes that are ubiquitous in (mouse) tissues [54, 55]. As long as tissues are alive and cell membranes intact, RNases cannot break down intracellular mRNAs. However, when membranes get permeabilized-e.g., due to freezing or natural degradation during tissue processing-RNases will have the opportunity to degrade mRNA [56]. Evidently, it is important to at least prevent ‘contamination’ of the tissues by exogenous RNases during tissue handling. When tissues, however, harbor high levels of endogenous RNases, protection against exogenous RNase may be insufficient to prevent RNA degradation. In postnatal lungs, a major part of the volume should be considered as ‘outside world’, where high amounts of RNases are present in all air spaces. Therefore, the endogenous RNase activity in lungs is about 5000 times higher than that of kidney or brain [57]. This explains the difference in integrity that was illustrated in the present study between identically treated LMD-isolated postnatal mouse lung tissue, which shows very poor RNA integrity, and brain tissue of the same mouse or from embryonic mouse lung, which are both sterile and reveal good RNA integrity. In the present study, the addition of RNase inhibitors (such as SuperaseIn®) to a maximum of the applied solutions strongly reduced damage by RNases in postnatal lungs. These solutions limit RNA degradation to a minimum during inevitable aqueous phases, and are certainly beneficial to downstream gene expression analysis [58–60].

Microdissection requires routinely prepared cryosections without coverslip, which greatly reduces the microscopic detail and compromises the ability to distinguish and isolate specific cell populations [56]. For the selective visualization of the cell types of interest, standard immunocytochemical staining protocols usually require several hours of incubation in aqueous media, resulting in significant RNA degradation ([61], own unpublished observations). In our lab, we recently adopted a GAD67-GFP mouse model, in which pulmonary NEBs can be characterized as GFP-fluorescent cell groups in the airway epithelium [8]. Since in this model the necessity of immunocytochemical staining for the selective identification of NEBs is eliminated, the GAD67-GFP mouse model was used for the presented gene expression studies of the NEB ME. The use of cryosections of the lungs of GAD67-GFP mice allows for the fast and unambiguous visualization of NEBs that are widely dispersed in the airway epithelium. It needs no further explanation that the final RNA integrity will greatly benefit from the possibility for direct selective LMD of the NEB ME, without long and complex preceding tissue handling.

In GAD67-GFP mice, GFP is expressed as a free protein in the cytoplasm and nucleus [8, 62]. Because of the relatively small size and the high solubility of GFP, fixation is required to prevent leakage of the protein and retain fluorescence after cryosectioning [63]. However, excessive fixation of the tissue prior to embedding prevents the efficient extraction of RNA and compromises RNA integrity [64]. 4% PF, which is routinely applied for a few hours to maintain a qualitative morphology of tissues for light microscopy, is known to considerably degrade mRNA macromolecules [57]. We therefore examined the lowest possible PF concentration and duration that allowed good visualization of fluorescent NEBs after cryosectioning. It was found that GFP-fluorescent NEBs could still be observed after a 5-min-fixation with 0.1% PF, a fixation procedure that appeared to have a very limited influence on RNA integrity.

Large-scale qPCR or PCR array studies require sufficient input mRNA, which is often challenging when using material obtained from a limited number of cells. It has been reported that the Ribo-SPIA pre-amplification method (e.g., WT-Ovation kit) offers great advantage to generate sufficient material for qPCR studies [65, 66]. Benefits are the constant yield of pre-amplified cDNA independent of the initial amount of mRNA in the sample, the preservation of differential gene expression after pre-amplification without inducing substantial bias, and the lack of co-amplification of contaminating genomic DNA [65]. In the present study, pre-amplification of mRNA from pooled NEB samples (starting concentrations of about 300 pg of total NEB RNA appeared to be sufficient) yielded several micrograms of cDNA, thereby creating a useful tool for future large-scale gene expression profiling of the NEB ME.

It is, however, important to keep in mind that the relative transcript abundance present in the amplified sample might differ from the initial mRNA sample [67]. Although low-abundance transcripts and sequence-specific differences in amplification efficiency may result in a technical variability, different studies report that specimen- and treatment-specific differences in gene expression will generally be more pronounced than changes due to technical variability. Biological differences in gene expression levels can indeed be uncovered using LMD-isolated samples and consequent amplification and qPCR [68, 69].

In our LMD samples of postnatal lungs, small differences in initial stability and consequent amplification of small amounts of RNA might certainly affect reproducibility due to parameters that cannot be controlled, at least in a subset of genes in the PCR arrays. Among other less favorable parameters is the fact that the general reference genes that are included in the arrays are not the most stable for our specimens, and that the quality of the specific primers for the panel of genes can hardly be controlled. Because it would be very laborious and expensive to achieve statistical significance for differences in gene expression by repeating the PCR arrays for a large group of animals, we argue for an approach that can much more easily deal with these problems. Potentially interesting genes that ‘pop-out’ of the arrays should in our opinion preferentially be validated and further studied in detail individually by qPCR, based on an independent set of custom designed primers and an optimized set of reference genes, chosen (GeNorm software) for their stability and specificity in the lung samples. Multiple qPCR runs of samples from different animals then allow for controllable and reproducible quantification of the expression of the selected genes of interest.

Altogether, analysis of the performed PCR arrays, control genes for sample purity, selected reference genes, and validation using a proof-of-concept gene (Dll3) have proven that the LMD protocol can be adequately fine-tuned to collect RNA samples of the NEB ME with a sufficiently good integrity for high-quality complex gene expression analysis.

The use of commercially available PCR arrays revealed a higher expression of a large number of stem cell-related genes in the NEB ME compared to CAE (about 30% with two-fold threshold and 14% with four-fold threshold). This observation is in line with general reviews on lung stem cells, suggesting that the NEB ME is one of the potential sources of stem cells that are dispersed along conducting airways [4, 38, 42]. In these reviews, especially CLCs are put forward to have stem cell characteristics [24, 52, 70–73]. In a model of severe airway injury induced by ablating all Clara cells with naphthalene [74], CLCs that do not express a specific cytochrome P450 enzyme can survive destruction and are apparently able to regenerate Clara cells and restore the airway epithelium.

In view of the proposed stem cell characteristics of the NEB ME, pulmonary neuroendocrine cells have been suggested to be a potential source for SCLC [45, 46, 75, 76]. Inactivation of Rb and Trp53, especially in CGRP-expressing airway epithelial cells, appeared to be sufficient for creating a SCLC model [47]. The tumor cells resemble neuroendocrine cells, expressing some characteristic markers, and have the potential to metastasize [47, 77].

The present study, in which several stem cell-related genes were expressed in the NEB ME, adds to the assumption that pulmonary NEBs could play an important role as a stem cell niche in postnatal lungs. Evidently, genes of the selected panel with a clearly up- or down-regulated expression in the NEB ME – compared to CAE-will need to be examined with the intention to fully characterize the potential significance of the postnatal NEB ME as a functional stem cell niche.

Of all the genes that were analyzed, Dll3 was found specifically in the NEB ME, while almost absent from CAE, resulting in the highest upregulation when PCR products were compared in arrays. In general, binding of Dll3-a transmembranous ligand-with a Notch receptor, will result in downstream activation of the pathway [78]. Our immunocytochemical staining confirmed the plasma membrane localization of Dll3 protein in NEB cells, suggesting a role for Notch signaling via Dll3 in the NEB ME of postnatal mouse lungs.

During development, mouse CLCs express the Notch intracellular domain [71], indicating that Notch signaling is involved in their embryonic cell-fate [71, 79, 80]. The current observation that Dll3 is expressed on the surface membrane of mouse PNECs, may reflect that PNECs express the ligand that is important for Notch signaling in the surrounding CLCs. This strengthens the belief that the NEB ME may also function as a stem cell niche in healthy postnatal lungs.

Pulmonary neuroendocrine cells express the transcription factor achaete scute homolog-1 (Ascl1; murine orthologue *Mash1*) during development [71, 81]. It has been proposed that Ascl1 can bind the Dll3 promoter and that Dll3 is therefore a target of transcription of Ascl1 [75, 82]. A recent study revealed that high-grade pulmonary neuroendocrine tumor-initiating cells in mice can be targeted with and also eradicated by a Dll3 antibody-drug conjugate. The induced SCLC cells apparently showed a high expression of Dll3 [75]. Dll3 can stimulate growth, migration and invasion of lung cancer cells [83, 84]. This observation is particularly interesting, since pulmonary neuroendocrine cells have been proposed as tumor-initiating cells for SCLC [46, 75, 85].

## Conclusions

The present study in postnatal mouse lungs showed that it is a realistic goal to obtain sufficient amounts of high-quality mRNA from very demanding specimens that need LMD and sample pooling. It should, however, be clear that special attention is needed for optimally conserving RNA integrity during every step of the, sometimes complex, protocols. In some cases, as illustrated by the present study of the pulmonary NEB ME in postnatal GAD67-GFP mice, it may be very useful to start from a model with genetically tagged cells of interest, because it significantly reduces manipulation steps. Our findings revealed that the use of very mild fixation, maximally reducing the time that the tissue is exposed to an aqueous environment, and inhibition of the endogenous RNase enzyme activity in all steps (and even before start) of the procedure, strongly enhance the integrity of the final mRNA output. The efforts to cope with the small sample size after LMD are described, together with the possibility to perform RNA amplification if necessary.

The new LMD model for isolation of GFP-fluorescent NEBs, and subsequent downstream gene expression analysis, represent a promising tool for further unraveling the molecular basis of NEB ME physiology in general, and its postnatal stem cell capacities in particular. The selective expression of Dll3 in postnatal mouse NEB cells, both at the gene expression and protein level, is certainly intriguing with respect to the cancer stem cell discussion. Dll3 has been chosen as a proof-of-concept gene, but many other genes attracted our attention in the performed PCR arrays, revealing both notably high and low expression levels. Main future perspective is to investigate in detail those genes with direct potential for boosting our knowledge on the functional significance of NEBs and their microenvironment in health and disease. One of the ongoing challenges is to extract from the data pool a specific marker panel for CLCs, which should allow selective targeting of these unique stem cells in the NEB ME without affecting the abundant Clara Cells in the general airway epithelium.

## Additional file

Additional file 1: Table S1. List of all genes that are represented in the performed RT^2^ Profiler™ PCR Arrays (Qiagen; see Table 2), arranged per array in alphabetical order. Included are the raw expression data (C(t) values, cut-off value 35) for each gene, both for pooled NEB ME and CAE samples. Data for the reference genes are listed first (shaded rows), followed by the specific genes for every array. (PDF 2030 kb)

## Abbreviations

Ascl1: Achaete scute homolog-1
CAE: Control airway epithelium
CCSP: Clara cell secretory protein
CGRP: Calcitonin gene-related peptide
CLC: Clara-like cell
Dll3: Delta-like ligand 3
eEF2: Eukaryotic translation elongation factor 2
Flt-1: FMS-like tyrosine kinase 1
GAD67: Glutamic acid decarboxylase 67
LMD: Laser microdissection
Mash1: Murine achaete scute homolog-1
ME: Microenvironment
MIQE: Minimum information for publication of q(RT)-PCR experiments
NEB: Neuroepithelial body
PD: Postnatal day
PET: Polyethylene terephthalate
PF: Paraformaldehyde
PNEC: Pulmonary neuroendocrine cell
qPCR: q(RT)-PCR: Quantitative (real-time) RT-PCR
RIN: RNA integrity number
Rpl38: Ribosomal protein L38
Rpl4: Ribosomal protein L4
RT-PCR: Reverse transcription polymerase chain reaction
SCLC: Small cell lung carcinoma
