# Supplementary material for: Selective gene expression analysis of the neuroepithelial body microenvironment in postnatal lungs with special interest for potential stem cell characteristics
Source: Respir Res. 2017 May 8;18:87. doi: 10.1186/s12931-017-0571-4 (PMC5422937; doi:10.1186/s12931-017-0571-4)
Supplement: Additional file 1: Table S1. — List of all genes that are represented in the performed RT2 Profiler™ PCR Arrays (Qiagen; see Table 2), arranged per array in alphabetical order. Included are the raw expression data (C(t) values, cut-off value 35) for each gene, both for pooled NEB ME and CAE samples. Data for the reference genes are listed first (shaded rows), followed by the specific genes for every array. (PDF 2030 kb) [file 12931_2017_571_MOESM1_ESM.pdf]

Table S1: List of all genes that are represented in the performed RT² Profiler™ PCR Arrays (Qiagen; see Table 2), arranged per array in alphabetical order. Included are the raw expression data (C(t) values; cut-off value 35) for each gene, both for pooled NEB ME and CAE samples. Data for the reference genes are listed first (shaded rows), followed by the specific genes for every array.

Cancer Stem Cells (PAMM-176Z)

Gene	Description	C(t) value CAE	C(t) value NEB ME
Actb	Actin, beta	22,01	23,2
B2m	Beta-2 microglobulin	23,47	24,54
Gapdh	Glyceraldehyde-3-phosphate dehydrogenase	22,93	23,94
Gusb	Glucuronidase, beta	25,85	26,48
Hsp90ab1	Heat shock protein 90 alpha (cytosolic), class B member 1	23,12	23,34
Abcb5	ATP-binding cassette, sub-family B (MDR/TAP), member 5	35	35
Abcg2	ATP-binding cassette, sub-family G (WHITE), member 2	30,36	33,82
Alcam	Activated leukocyte cell adhesion molecule	24,56	24,83
Aldh1a1	Aldehyde dehydrogenase family 1, subfamily A1	21,97	22,63
Atm	Ataxia telangiectasia mutated homolog (human)	30,83	30,33
Atxn1	Ataxin 1	27,12	28,29
Axl	AXL receptor tyrosine kinase	26,87	29,43
Bmi1	Bmi1 polycomb ring finger oncogene	26,76	26,9
Bmp7	Bone morphogenetic protein 7	29,04	28,91
Cd24a	CD24a antigen	21,74	22,78
Cd34	CD34 antigen	25,42	28
Cd38	CD38 antigen	27,72	29,13
Cd44	CD44 antigen	26,21	26,66
Chek1	Checkpoint kinase 1 homolog (S. pombe)	35	35
Dach1	Dachshund 1 (Drosophila)	29,89	32,27
Ddr1	Discoidin domain receptor family, member 1	26,91	27,3
Dkk1	Dickkopf homolog 1 (Xenopus laevis)	35	35
Dll1	Delta-like 1 (Drosophila)	33,96	33,65
Dll4	Delta-like 4 (Drosophila)	30,52	29,8
Dnmt1	DNA methyltransferase (cytosine-5) 1	27,85	29
Egf	Epidermal growth factor	35	35
Eng	Endoglin	26,69	29,52
Epcam	Epithelial cell adhesion molecule	26,58	24,75
Erbb2	V-erb-b2 erythroblastic leukemia viral oncogene homolog 2	28,23	27,17
Etfa	Electron transferring flavoprotein, alpha polypeptide	24,04	25,27
Fgfr2	Fibroblast growth factor receptor 2	25,77	25,7
Flot2	Flotillin 2	25,92	26,61
Foxa2	Forkhead box A2	27,34	26,46
Foxp1	Forkhead box P1	26,31	26,28
Fzd7	Frizzled homolog 7 (Drosophila)	28,79	29,94
Gata3	GATA binding protein 3	32,34	31,89
Gsk3b	Glycogen synthase kinase 3 beta	26,2	26,34
Hdac1	Histone deacetylase 1	26,2	26,18
Id1	Inhibitor of DNA binding 1	24,92	26,08
Ikbkb	Inhibitor of kappaB kinase beta	28,55	27,85
Itga2	Integrin alpha 2	29,99	29,78
Itga4	Integrin alpha 4	30,03	30,27

Itga6	Integrin alpha 6	27,89	28,89
Itgb1	Integrin beta 1 (fibronectin receptor beta)	23,44	24,11
Jag1	Jagged 1	27,57	27,78
Jak2	Janus kinase 2	26,26	27,03
Kit	Kit oncogene	29,24	28,92
Kitl	Kit ligand	25	26,06
Klf17	Kruppel-like factor 17	35	35
Klf4	Kruppel-like factor 4 (gut)	24,98	25,79
Lats1	Large tumor suppressor	29,03	29,24
Lin28a	Lin-28 homolog A (C. elegans)	35	35
Lin28b	Lin-28 homolog B (C. elegans)	35	35
Maml1	Mastermind like 1 (Drosophila)	28,08	30,02
Mertk	C-mer proto-oncogene tyrosine kinase	29,46	30,1
Ms4a1	Membrane-spanning 4-domains, subfamily A, member 1	30,43	35
Muc1	Mucin 1, transmembrane	24,78	24,98
Myc	Myelocytomatosis oncogene	30,58	31,72
Mycn	V-myc myelocytomatosis viral related oncogene, neuroblastoma derived	35	35
Nanog	Nanog homeobox	35	32,06
Nfkb1	Nuclear factor of kappa light polypeptide gene enhancer in B-cells 1	26,08	27,02
Nos2	Nitric oxide synthase 2, inducible	35	35
Notch1	Notch gene homolog 1 (Drosophila)	29,75	32,03
Notch2	Notch gene homolog 2 (Drosophila)	29,82	29,15
Pecam1	Platelet/endothelial cell adhesion molecule 1	26,53	29,64
Plat	Plasminogen activator, tissue	27,67	28,2
Plaur	Plasminogen activator, urokinase receptor	27,53	28,22
Pou5f1	POU domain, class 5, transcription factor 1	35	35
Prom1	Prominin 1	26,77	26,92
Ptch1	Patched homolog 1	30,85	30,25
Ptprc	Protein tyrosine phosphatase, receptor type, C	28,92	35
Sav1	Salvador homolog 1 (Drosophila)	25,55	26,11
Sirt1	Sirtuin 1 (silent mating type information regulation 2, homolog) 1	30,23	28,5
Smo	Smoothened homolog (Drosophila)	30,48	32,95
Snai1	Snail homolog 1 (Drosophila)	33,95	33,88
Snai2	Snail homolog 2 (Drosophila)	29,1	30,55
Sox2	SRY-box containing gene 2	27,46	26,54
Stat3	Signal transducer and activator of transcription 3	25,57	25,3
Taz	Tafazzin	27,22	28,8
Tgfbr1	Transforming growth factor, beta receptor I	26,25	26,66
Thy1	Thymus cell antigen 1, theta	29,46	31,81
Twist1	Twist homolog 1 (Drosophila)	31,8	32,86
Twist2	Twist homolog 2 (Drosophila)	28,06	31,48
Wee1	WEE 1 homolog 1 (S. pombe)	27,57	28,55
Wnt1	Wingless-related MMTV integration site 1	35	35
Wwc1	WW, C2 and coiled-coil domain containing 1	27,21	27,55
Yap1	Yes-associated protein 1	25,81	26,56
Zeb1	Zinc finger E-box binding homeobox 1	29,44	28,97
Zeb2	Zinc finger E-box binding homeobox 2	26,12	28,24

Growth Factors (PAMM-041Z)

Gene	Description	C(t) value CAE	NEB ME
Actb	Actin, beta	19,16	18,81
B2m	Beta-2 microglobulin	21	21,03
Gapdh	Glyceraldehyde-3-phosphate dehydrogenase	21,11	21,05
Gusb	Glucuronidase, beta	23,23	22,85
Hsp90ab1	Heat shock protein 90 alpha (cytosolic), class B member 1	20,34	19,77
Amh	Anti-Mullerian hormone	33,51	32,55
Artn	Artemin	27,53	26,04
Bdnf	Brain derived neurotrophic factor	27,87	29
Bmp1	Bone morphogenetic protein 1	23,34	23,31
Bmp10	Bone morphogenetic protein 10	35	35
Bmp2	Bone morphogenetic protein 2	27,49	24,31
Bmp3	Bone morphogenetic protein 3	26,57	26,22
Bmp4	Bone morphogenetic protein 4	23,58	23,91
Bmp5	Bone morphogenetic protein 5	23,26	22,99
Bmp6	Bone morphogenetic protein 6	24,58	25,95
Bmp7	Bone morphogenetic protein 7	26,21	24,24
Bmp8a	Bone morphogenetic protein 8a	30,07	29,48
Bmp8b	Bone morphogenetic protein 8b	29,04	28,17
Csf1	Colony stimulating factor 1 (macrophage)	25,87	25,72
Csf2	Colony stimulating factor 2 (granulocyte-macrophage)	30,43	32,43
Csf3	Colony stimulating factor 3 (granulocyte)	35	35
Cxcl1	Chemokine (C-X-C motif) ligand 1	32,08	33,85
Cxcl12	Chemokine (C-X-C motif) ligand 12	22,99	24,15
Egf	Epidermal growth factor	29,51	30,62
Ereg	Epiregulin	29,26	28,13
Fgf1	Fibroblast growth factor 1	22,88	23,31
Fgf10	Fibroblast growth factor 10	26,73	27,21
Fgf11	Fibroblast growth factor 11	26,99	26,8
Fgf13	Fibroblast growth factor 13	26,25	24
Fgf14	Fibroblast growth factor 14	29,26	23,64
Fgf15	Fibroblast growth factor 15	31,98	33,06
Fgf17	Fibroblast growth factor 17	35	32,25
Fgf18	Fibroblast growth factor 18	26,1	26,56
Fgf2	Fibroblast growth factor 2	25,78	25,71
Fgf22	Fibroblast growth factor 22	35	31,31
Fgf3	Fibroblast growth factor 3	30,78	34,12
Fgf4	Fibroblast growth factor 4	35	35
Fgf5	Fibroblast growth factor 5	31,07	25,49
Fgf6	Fibroblast growth factor 6	35	35
Fgf7	Fibroblast growth factor 7	24,34	24,1
Fgf8	Fibroblast growth factor 8	35	35
Fgf9	Fibroblast growth factor 9	31,02	30,46
Figf	C-fos induced growth factor	24,09	24,43
Gdf10	Growth differentiation factor 10	26,58	26,09
Gdf11	Growth differentiation factor 11	27,06	26,06
Gdf5	Growth differentiation factor 5	28,15	27,69
Gdnf	Glial cell line derived neurotrophic factor	30,03	27,94

Hgf	Hepatocyte growth factor	25,93	23,81
Igf1	Insulin-like growth factor 1	23,55	23,67
Igf2	Insulin-like growth factor 2	27,13	23,66
Il11	Interleukin 11	31,72	30,17
Il12a	Interleukin 12A	31,02	30,08
Il18	Interleukin 18	26,88	26,8
Il1a	Interleukin 1 alpha	27,8	28,28
Il1b	Interleukin 1 beta	28,34	27,75
Il2	Interleukin 2	35	32,48
Il3	Interleukin 3	35	35
Il4	Interleukin 4	30,68	28,48
Il6	Interleukin 6	30,98	31,96
Il7	Interleukin 7	26,47	26,69
Inha	Inhibin alpha	25,81	24,28
Inhba	Inhibin beta-A	26,19	25,5
Inhbb	Inhibin beta-B	25,64	25,13
Kitl	Kit ligand	21,45	21,88
Lefty1	Left right determination factor 1	25,78	26,24
Lefty2	Left-right determination factor 2	32,06	31,92
Lep	Leptin	29,24	35
Lif	Leukemia inhibitory factor	26,84	26,43
Mdk	Midkine	23,48	23,59
Mstn	Myostatin	35	31,02
Ngf	Nerve growth factor	28,04	26,57
Nodal	Nodal	35	35
Ntf3	Neurotrophin 3	25,44	25,15
Ntf5	Neurotrophin 5	27,26	27,08
Pdgfa	Platelet derived growth factor, alpha	23,64	22,95
Pgf	Placental growth factor	29,07	27,14
Rabep1	Rabaptin, RAB GTPase binding effector protein 1	23,51	23,07
S100a6	S100 calcium binding protein A6 (calcyclin)	18,81	18,55
Spp1	Secreted phosphoprotein 1	27,06	22,68
Tdgf1	Teratocarcinoma-derived growth factor 1	35	34,95
Tff1	Trefoil factor 1	27,78	26,98
Tgfa	Transforming growth factor alpha	25,79	24,99
Tgfb1	Transforming growth factor, beta 1	23,21	23,75
Tgfb2	Transforming growth factor, beta 2	25,64	24,8
Tgfb3	Transforming growth factor, beta 3	24,03	23,22
Vegfa	Vascular endothelial growth factor A	22,03	22,16
Vegfb	Vascular endothelial growth factor B	24,93	25,16
Vegfc	Vascular endothelial growth factor C	27,06	26,84
Zfp91	Zinc finger protein 91	22,34	21,71

Hedgehog Pathway (PAMM078Z)

Gene	Description	C(t) value CAE	NEB ME
Actb	Actin, beta	20,59	24,11
B2m	Beta-2 microglobulin	23,67	26,7
Gapdh	Glyceraldehyde-3-phosphate dehydrogenase	22,11	25,3
Gusb	Glucuronidase, beta	24,16	27
Hsp90ab1	Heat shock protein 90 alpha (cytosolic), class B member 1	21,13	24,13
Bcl2	B-cell leukemia/lymphoma 2	29,51	31,7
Bmp2	Bone morphogenetic protein 2	30,04	30,68
Bmp4	Bone morphogenetic protein 4	25,06	28,62
Bmp5	Bone morphogenetic protein 5	26,2	28,78
Bmp6	Bone morphogenetic protein 6	27,45	31,06
Bmp7	Bone morphogenetic protein 7	26,53	27,5
Bmp8a	Bone morphogenetic protein 8a	30,32	35
Bmp8b	Bone morphogenetic protein 8b	31,05	31,7
Boc	Biregional Cdon-binding protein	26,2	29,18
Btrc	Beta-transducin repeat containing protein	27,59	28,97
Cdon	Cell adhesion molecule-related/down-regulated by oncogenes	27,99	32,01
Csnk1a1	Casein kinase 1, alpha 1	22,6	24,88
Csnk1e	Casein kinase 1, epsilon	24,68	26,64
Ctnnb1	Catenin (cadherin associated protein), beta 1	21,75	24,53
Dhh	Desert hedgehog	32,93	35
Disp1	Dispatched homolog 1 (Drosophila)	29,76	31,66
Disp2	Dispatched homolog 2 (Drosophila)	30,08	28,34
Erbb4	V-erb-a erythroblastic leukemia viral oncogene homolog 4 (avian)	33,35	35
Fat4	FAT tumor suppressor homolog 4 (Drosophila)	27,95	31,86
Fbxw11	F-box and WD-40 domain protein 11	24,98	27,24
Fgf9	Fibroblast growth factor 9	33,53	33,61
Fgfr3	Fibroblast growth factor receptor 3	24,45	27,14
Fkbp8	FK506 binding protein 8	23,76	26,97
Foxe1	Forkhead box E1	35	35
Frmd6	FERM domain containing 6	28,83	33,01
Gas1	Growth arrest specific 1	26,07	31,71
Gli1	GLI-Kruppel family member GLI1	27,62	31,65
Gli2	GLI-Kruppel family member GLI2	29,92	33,71
Gli3	GLI-Kruppel family member GLI3	28,85	31,23
Grem1	Gremlin 1	29,13	30,47
Gsk3b	Glycogen synthase kinase 3 beta	24,81	25,73
Hhat	Hedgehog acyltransferase	30,03	31,9
Hhip	Hedgehog-interacting protein	23,61	25,78
Ihh	Indian hedgehog	32,9	35
Kctd11	Potassium channel tetramerisation domain containing 11	25,95	29,57
Lats1	Large tumor suppressor	28,08	29,97
Lats2	Large tumor suppressor 2	25,06	28,99
Lrp2	Low density lipoprotein receptor-related protein 2	32,57	35
Mapk1	Mitogen-activated protein kinase 1	22,93	25,32
Mob1b	MOB1, Mps One Binder kinase activator-like 1A (yeast)	24,86	26,84
Mtss1	Metastasis suppressor 1	30,24	30,49
Nf2	Neurofibromatosis 2	25,01	27,1

Npc1	Niemann Pick type C1	26,14	29,47
Numb	Numb gene homolog (Drosophila)	27,46	30,97
Otx2	Orthodenticle homolog 2 (Drosophila)	35	35
Prkaca	Protein kinase, cAMP dependent, catalytic, alpha	25,2	27,98
Prkacb	Protein kinase, cAMP dependent, catalytic, beta	24,8	27
Ptch1	Patched homolog 1	26,9	28,98
Ptch2	Patched homolog 2	30,24	35
Ptchd2	Patched domain containing 2	35	35
Ptchd3	Patched domain containing 3	29,79	35
Rab23	RAB23, member RAS oncogene family	25,23	27,27
Runx2	Runt related transcription factor 2	31,05	35
Sfrp1	Secreted frizzled-related protein 1	29,81	35
Shh	Sonic hedgehog	24,21	26,78
Shox2	Short stature homeobox 2	31,81	34,44
Smo	Smoothened homolog (Drosophila)	28,49	32,98
Stk3	Serine/threonine kinase 3 (Ste20, yeast homolog)	25,15	26,49
Stk36	Serine/threonine kinase 36 (fused homolog, Drosophila)	26,15	28,69
Sufu	Suppressor of fused homolog (Drosophila)	26,45	28,85
Trp53	Transformation related protein 53	25,89	28,62
Vegfa	Vascular endothelial growth factor A	23,66	25,9
Wif1	Wnt inhibitory factor 1	24,18	26,61
Wnt1	Wingless-related MMTV integration site 1	34,72	35
Wnt10a	Wingless related MMTV integration site 10a	35	35
Wnt10b	Wingless related MMTV integration site 10b	29,98	31,86
Wnt11	Wingless-related MMTV integration site 11	24,44	27,06
Wnt16	Wingless-related MMTV integration site 16	29,59	29,56
Wnt2	Wingless-related MMTV integration site 2	31,58	35
Wnt2b	Wingless related MMTV integration site 2b	31,43	34,79
Wnt3	Wingless-related MMTV integration site 3	35	35
Wnt3a	Wingless-related MMTV integration site 3A	34,08	35
Wnt4	Wingless-related MMTV integration site 4	28,49	30,51
Wnt5a	Wingless-related MMTV integration site 5A	25,58	28,07
Wnt5b	Wingless-related MMTV integration site 5B	27,83	31,41
Wnt6	Wingless-related MMTV integration site 6	28,97	31,31
Wnt7a	Wingless-related MMTV integration site 7A	35	35
Wnt7b	Wingless-related MMTV integration site 7B	31,2	35
Wnt8a	Wingless-related MMTV integration site 8A	35	35
Wnt8b	Wingless related MMTV integration site 8b	30,93	35
Wnt9a	Wingless-type MMTV integration site 9A	33,42	35
Wnt9b	Wingless-type MMTV integration site 9B	35	35
Zic1	Zinc finger protein of the cerebellum 1	35	35
Zic2	Zinc finger protein of the cerebellum 2	35	35

Hippo Signaling Pathway (PAMM-172Z)

Gene	Description	C(t) value CAE	NEB ME
Actb	Actin, beta	19,55	21,31
B2m	Beta-2 microglobulin	21,76	23,27
Gapdh	Glyceraldehyde-3-phosphate dehydrogenase	20,98	22,28
Gusb	Glucuronidase, beta	23,5	25,08
Hsp90ab1	Heat shock protein 90 alpha (cytosolic), class B member 1	20,82	21,89
Actg1	Actin, gamma, cytoplasmic 1	19,51	20,89
Amot	Angiomotin	29,18	29,76
Amotl1	Angiomotin-like 1	25,95	27,51
Amotl2	Angiomotin-like 2	22,73	23,66
Casp3	Caspase 3	25,95	27,85
Ccne1	Cyclin E1	28,55	31,29
Ccne2	Cyclin E2	25,91	28,26
Crb1	Crumbs homolog 1 (Drosophila)	30,44	31,79
Crb2	Crumbs homolog 2 (Drosophila)	32,08	31,27
Crb3	Crumbs homolog 3 (Drosophila)	24,15	25,07
Csnk1d	Casein kinase 1, delta	23,79	25,09
Csnk1e	Casein kinase 1, epsilon	24,06	24,79
Dchs1	Dachsous 1 (Drosophila)	27,18	28,67
Dchs2	Dachsous 2 (Drosophila)	28,82	29,11
Diap2	Diaphanous homolog 2 (Drosophila)	25,47	27,72
Dlg1	Discs, large homolog 1 (Drosophila)	23,24	24,58
Dvl2	Dishevelled 2, dsh homolog (Drosophila)	26,8	27,92
Fat1	FAT tumor suppressor homolog 1 (Drosophila)	23,48	23,64
Fat2	FAT tumor suppressor homolog 2 (Drosophila)	30,13	29,07
Fat3	FAT tumor suppressor homolog 3 (Drosophila)	27,48	32,21
Fat4	FAT tumor suppressor homolog 4 (Drosophila)	27,06	28,22
Fjx1	Four jointed box 1 (Drosophila)	29,12	33,65
Gpc5	Glypican 5	30,25	35
Hipk2	Homeodomain interacting protein kinase 2	26,81	27,49
Hmcn1	Hemicentin 1	25,46	27,63
Ajuba	Ajuba	26,06	26,89
Lats1	Large tumor suppressor	26,34	26,75
Lats2	Large tumor suppressor 2	24,52	26,78
Limd1	LIM domains containing 1	23,96	25,49
Lix1l	Lix1-like	24,31	26,48
Llgl1	Lethal giant larvae homolog 1 (Drosophila)	26,59	28,18
Llgl2	Lethal giant larvae homolog 2 (Drosophila)	28,65	29,67
Lpp	LIM domain containing preferred translocation partner in lipoma	23,77	25,08
Mapk10	Mitogen-activated protein kinase 10	34,3	27,74
Meis1	Meis homeobox 1	28,48	31,47
Mob1b	MOB1, Mps One Binder kinase activator-like 1A (yeast)	23,54	24,28
Mob1a	MOB1, Mps One Binder kinase activator-like 1B (yeast)	24,74	26,81
Mpdz	Multiple PDZ domain protein	25,57	26,6
Mpp5	Membrane protein, palmitoylated 5 (MAGUK p55 subfamily member 5)	24,67	26,23
Mst1	Macrophage stimulating 1 (hepatocyte growth factor-like)	25,61	27,03
Myc	Myelocytomatosis oncogene	28,72	29,15
Nf2	Neurofibromatosis 2	23,88	25,54

Nphp4	Nephronophthisis 4 (juvenile) homolog (human)	25,89	26,56
Pard3	Par-3 (partitioning defective 3) homolog (C. elegans)	24,2	25,26
Pard6g	Par-6 partitioning defective 6 homolog gamma (C. elegans)	26,19	27,28
Poteg	POTE ankyrin domain family, member G	31,43	35
Ppp2cb	Protein phosphatase 2 (formerly 2A), catalytic subunit β	22,64	24,06
Ppp2r1a	Protein phosphatase 2 (formerly 2A), regulatory subunit A (PR 65) α	22,33	23,03
Ppp2r2d	Protein phosphatase 2, regulatory subunit B δ	24,14	25,26
Prkci	Protein kinase C, iota	24,34	26,04
Prkcz	Protein kinase C, zeta	23,88	24,77
Ptpn14	Protein tyrosine phosphatase, non-receptor type 14	28,43	29,84
Rassf2	Ras association (RalGDS/AF-6) domain family member 2	25,8	27,09
Rassf4	Ras association (RalGDS/AF-6) domain family member 4	24,98	26,6
Rere	Arginine glutamic acid dipeptide (RE) repeats	29,03	29,09
Sav1	Salvador homolog 1 (Drosophila)	22,8	24,14
Scrib	Scribbled homolog (Drosophila)	28,92	29,9
Smad1	MAD homolog 1 (Drosophila)	24,85	25,85
Stk3	Serine/threonine kinase 3 (Ste20, yeast homolog)	23,9	24,84
Stk4	Serine/threonine kinase 4	25,21	26,21
Taok1	TAO kinase 1	23,95	25,49
Taok2	TAO kinase 2	29,46	31,15
Taok3	TAO kinase 3	24,3	25,08
Taz	Tafazzin	24,19	25,52
Tead1	TEA domain family member 1	24,68	25,6
Tead2	TEA domain family member 2	24,9	26,47
Tead3	TEA domain family member 3	26,75	28,14
Tead4	TEA domain family member 4	27,24	29,22
Tjp1	Tight junction protein 1	25,48	26,2
Tjp2	Tight junction protein 2	25,17	26,44
Trp63	Transformation related protein 63	31,31	35
Tshz1	Teashirt zinc finger family member 1	25,8	27,49
Tshz2	Teashirt zinc finger family member 2	24,51	25,29
Tshz3	Teashirt zinc finger family member 3	26,23	27,97
Wnt1	Wingless-related MMTV integration site 1	33,75	35
Wtip	WT1-interacting protein	24,68	26,35
Wwc1	WW, C2 and coiled-coil domain containing 1	24,11	25,69
Wwtr1	WW domain containing transcription regulator 1	22,93	24,69
Yap1	Yes-associated protein 1	23,54	24,59
Ywhab	Tyrosine 3-/tryptophan 5-monooxygenase activation protein β	23,47	25,02
Ywhae	Tyrosine 3-/tryptophan 5-monooxygenase activation protein ε	20,49	21,76
Ywhaq	Tyrosine 3-/tryptophan 5-monooxygenase activation protein θ	25,89	26,59
Ywhaz	Tyrosine 3-/tryptophan 5-monooxygenase activation protein ζ	20,92	22,23
Zdhhc18	Zinc finger, DHHC domain containing 18	24,7	26,22

Notch Signaling Pathway (PAMM-059Z)

Gene	Description	C(t) value CAE	NEB ME
Actb	Actin, beta	20,54	22,71
B2m	Beta-2 microglobulin	22,73	25,43
Gapdh	Glyceraldehyde-3-phosphate dehydrogenase	22,53	24,22
Gusb	Glucuronidase, beta	24,69	26,52
Hsp90ab1	Heat shock protein 90 alpha (cytosolic), class B member 1	21,7	22,88
Adam10	A disintegrin and metallopeptidase domain 10	25,99	27,64
Adam17	A disintegrin and metallopeptidase domain 17	26,61	27,52
Aes	Amino-terminal enhancer of split	21,17	22,64
Axin1	Axin 1	26,48	28,18
Cbl	Casitas B-lineage lymphoma	26,83	27,64
Ccnd1	Cyclin D1	24,01	24,95
Ccne1	Cyclin E1	30,57	32,24
Cd44	CD44 antigen	25,54	25,79
Cdkn1a	Cyclin-dependent kinase inhibitor 1A (P21)	24,59	26,31
Cflar	CASP8 and FADD-like apoptosis regulator	25,15	26,8
Chuk	Conserved helix-loop-helix ubiquitous kinase	28,04	32,19
Ctnnb1	Catenin (cadherin associated protein), beta 1	21,85	23,82
Dll1	Delta-like 1 (Drosophila)	31,85	33,83
Dll3	Delta-like 3 (Drosophila)	35	28,42
Dll4	Delta-like 4 (Drosophila)	27,62	28,59
Dtx1	Deltex 1 homolog (Drosophila)	29,13	31,49
Ep300	E1A binding protein p300	28,89	30,45
Erbb2	V-erb-b2 avian erythroblastic leukemia viral oncogene homolog 2	25,77	27,16
Figf	C-fos induced growth factor	25,97	29,2
Fos	FBJ osteosarcoma oncogene	26,9	28,13
Fosl1	Fos-like antigen 1	35	35
Fzd2	Frizzled homolog 2 (Drosophila)	27,9	29,23
Fzd3	Frizzled homolog 3 (Drosophila)	28,13	29,2
Fzd4	Frizzled homolog 4 (Drosophila)	27,87	30,65
Fzd5	Frizzled homolog 5 (Drosophila)	27,86	30,34
Fzd7	Frizzled homolog 7 (Drosophila)	26,91	29,12
Gli1	GLI-Kruppel family member GLI1	31,21	35
Gsk3b	Glycogen synthase kinase 3 beta	25,05	25,21
Hes1	Hairy and enhancer of split 1 (Drosophila)	24,53	25,69
Hes5	Hairy and enhancer of split 5 (Drosophila)	35	35
Hey1	Hairy/enhancer-of-split related with YRPW motif 1	25,51	27,02
Hey2	Hairy/enhancer-of-split related with YRPW motif 2	30,21	33,76
Heyl	Hairy/enhancer-of-split related with YRPW motif-like	26,6	27,73
Hoxb4	Homeobox B4	35	35
hr	Hairless	29,46	30,76
Id1	Inhibitor of DNA binding 1	24,47	26,99
Ifng	Interferon gamma	35	35
Il17b	Interleukin 17B	30,7	30,95
Il2ra	Interleukin 2 receptor, alpha chain	32,24	35
Il6st	Interleukin 6 signal transducer	24,16	25,45
Jag1	Jagged 1	25,91	26,7
Jag2	Jagged 2	27,95	29,08

Krt1	Keratin 1	31,88	31,8
Lfng	LFNG O-fucosylpeptide 3-beta-N-acetylglucosaminyltransferase	25,13	26,12
Lmo2	LIM domain only 2	25,25	27,29
Lor	Loricrin	35	31,03
Lrp5	Low density lipoprotein receptor-related protein 5	26,42	28,47
Maml1	Mastermind like 1 (Drosophila)	28,85	31,14
Maml2	Mastermind like 2 (Drosophila)	31,44	35
Mfng	MFNG O-fucosylpeptide 3-beta-N-acetylglucosaminyltransferase	27,72	27,52
Mmp7	Matrix metallopeptidase 7	35	35
Ncor2	Nuclear receptor co-repressor 2	30,68	30,75
Ncstn	Nicastrin	25,78	27,51
Neurl1a	Neuralized homolog 1A (Drosophila)	28	28,24
Nfkb1	Nuclear factor of κ light polypeptide gene enhancer in B-cells 1	25,13	26,92
Nfkb2	Nuclear factor of κ light polypeptide gene enhancer in B-cells 2	29,04	30,9
Notch1	Notch gene homolog 1 (Drosophila)	29,26	31,44
Notch2	Notch gene homolog 2 (Drosophila)	28,06	29,65
Notch3	Notch gene homolog 3 (Drosophila)	27,47	28,57
Notch4	Notch gene homolog 4 (Drosophila)	27,57	28,95
Nr4a2	Nuclear receptor subfamily 4, group A, member 2	32,33	32,64
Numb	Numb gene homolog (Drosophila)	27,46	28,5
Pax5	Paired box gene 5	31,9	31,04
Pofut1	Protein O-fucosyltransferase 1	29,71	32,47
Pparg	Peroxisome proliferator activated receptor gamma	26,66	28,32
Psen1	Presenilin 1	25,63	27,95
Psen2	Presenilin 2	29,12	30,71
Psenen	Presenilin enhancer 2 homolog (C. elegans)	22,66	24,68
Ptcra	Pre T-cell antigen receptor alpha	32,64	33,11
Rbpjl	Recombination signal binding protein for immunoglobulin-κ J region-like	29,29	35
Rfng	RFNG O-fucosylpeptide 3-beta-N-acetylglucosaminyltransferase	26,97	27,96
Runx1	Runt related transcription factor 1	23,95	25,11
Sel1l	Sel-1 suppressor of lin-12-like (C. elegans)	26,47	25,47
Shh	Sonic hedgehog	26,74	27,72
Smo	Smoothened homolog (Drosophila)	28,52	30,69
Snw1	SNW domain containing 1	25,13	26,79
Stat6	Signal transducer and activator of transcription 6	29,97	33
Stil	Scl/Tal1 interrupting locus	27,74	28,95
Sufu	Suppressor of fused homolog (Drosophila)	27,2	28,98
Supt6	Suppressor of Ty 6 homolog (S. cerevisiae)	29,95	30,55
Tle1	Transducin-like enhancer of split 1, homolog of Drosophila E(spl)	26,85	27,28
Wisp1	WNT1 inducible signaling pathway protein 1	27,91	30,55
Wnt11	Wingless-related MMTV integration site 11	25,59	28,14
Zic2	Zinc finger protein of the cerebellum 2	35	35

Signal Transduction Pathway Finder (PAMM-014Z)

Gene	Description	C(t) value CAE	NEB ME
Actb	Actin, beta	20,23	21,8
B2m	Beta-2 microglobulin	21,84	22,87
Gapdh	Glyceraldehyde-3-phosphate dehydrogenase	22,03	23,17
Gusb	Glucuronidase, beta	24,51	25,78
Hsp90ab1	Heat shock protein 90 alpha (cytosolic), class B member 1	20,75	21,61
Acsl3	Acyl-CoA synthetase long-chain family member 3	24,18	25,75
Acsl4	Acyl-CoA synthetase long-chain family member 4	26,3	26,7
Acsl5	Acyl-CoA synthetase long-chain family member 5	23,07	23,64
Adm	Adrenomedullin	30,28	35
Arnt	Aryl hydrocarbon receptor nuclear translocator	25,6	25,63
Atf4	Activating transcription factor 4	24,45	25,13
Axin2	Axin2	25,57	25,26
Bax	Bcl2-associated X protein	23,73	24,79
Bbc3	BCL2 binding component 3	28,97	28,61
Bcl2	B-cell leukemia/lymphoma 2	28,56	29
Bcl2a1a	B-cell leukemia/lymphoma 2 related protein A1a	27,52	33,11
Bcl2l1	Bcl2-like 1	24,3	24,61
Birc3	Baculoviral IAP repeat-containing 3	30,21	31,72
Bmp2	Bone morphogenetic protein 2	29,24	26,58
Bmp4	Bone morphogenetic protein 4	25,32	27,09
Btg2	B-cell translocation gene 2, anti-proliferative	23,13	23,33
Car9	Carbonic anhydrase 9	29,17	29,86
Ccl5	Chemokine (C-C motif) ligand 5	32,11	31,31
Ccnd1	Cyclin D1	22,6	22,59
Ccnd2	Cyclin D2	22,79	22,21
Cdkn1a	Cyclin-dependent kinase inhibitor 1A (P21)	23,84	26,18
Cdkn1b	Cyclin-dependent kinase inhibitor 1B	23,98	24,09
Cebpd	CCAAT/enhancer binding protein (C/EBP), delta	23,72	25,54
Cpt2	Carnitine palmitoyltransferase 2	25,59	26,54
Csf1	Colony stimulating factor 1 (macrophage)	27,28	28,74
Dab2	Disabled homolog 2 (Drosophila)	26,66	28,02
Egfr	Epidermal growth factor receptor	27,78	25,93
Emp1	Epithelial membrane protein 1	23,69	25,32
Epo	Erythropoietin	35	35
Fabp1	Fatty acid binding protein 1, liver	30,8	35
Fas	Fas (TNF receptor superfamily member 6)	24,63	25,88
Fcer2a	Fc receptor, IgE, low affinity II, alpha polypeptide	35	28,33
Fosl1	Fos-like antigen 1	35	35
Fth1	Ferritin heavy chain 1	17,46	18,5
Gadd45a	Growth arrest and DNA-damage-inducible 45 alpha	27,61	28,05
Gadd45b	Growth arrest and DNA-damage-inducible 45 beta	27,51	27,44
Gata3	GATA binding protein 3	32,63	35
Gclc	Glutamate-cysteine ligase, catalytic subunit	23,01	24,55
Gclm	Glutamate-cysteine ligase, modifier subunit	23	24,14
Gsr	Glutathione reductase	23,81	25,68
Herpud1	Homocysteine-, ER stress-inducible, ubiquitin-like domain member 1	26,62	27,61
Hes1	Hairy and enhancer of split 1 (Drosophila)	22,94	23,82

Hes5	Hairy and enhancer of split 5 (Drosophila)	35	35
Hey1	Hairy/enhancer-of-split related with YRPW motif 1	25,29	26,13
Hey2	Hairy/enhancer-of-split related with YRPW motif 2	35	34,27
Heyl	Hairy/enhancer-of-split related with YRPW motif-like	26,05	27,77
Hmox1	Heme oxygenase (decycling) 1	29,51	29,97
Icam1	Intercellular adhesion molecule 1	27,61	27,35
Id1	Inhibitor of DNA binding 1	24,09	24,96
Ifng	Interferon gamma	35	35
Ifrd1	Interferon-related developmental regulator 1	22,95	24,21
Irf1	Interferon regulatory factor 1	24,18	24,88
Jag1	Jagged 1	25,34	25,07
Ldha	Lactate dehydrogenase A	21,97	23,6
Lfng	LFNG O-fucosylpeptide 3-beta-N-acetylglucosaminyltransferase	24,33	24,69
Lrg1	Leucine-rich alpha-2-glycoprotein 1	27,79	32,96
Mcl1	Myeloid cell leukemia sequence 1	22,93	23,9
Mmp7	Matrix metallopeptidase 7	35	35
Myc	Myelocytomatosis oncogene	29,08	28,53
Notch1	Notch gene homolog 1 (Drosophila)	27,96	30,19
Nqo1	NAD(P)H dehydrogenase, quinone 1	27,73	29,06
Olr1	Oxidized low density lipoprotein (lectin-like) receptor 1	30,35	35
Pcna	Proliferating cell nuclear antigen	24,92	25,21
Ppard	Peroxisome proliferator activator receptor delta	28,77	29,07
Ptch1	Patched homolog 1	28,09	27,1
Rb1	Retinoblastoma 1	25,54	25,23
Serpine1	Serine (or cysteine) peptidase inhibitor, clade E, member 1	28,56	28,44
Slc27a4	Solute carrier family 27 (fatty acid transporter), member 4	26,49	27,17
Slc2a1	Solute carrier family 2 (facilitated glucose transporter), member 1	25,52	26,5
Socs3	Suppressor of cytokine signaling 3	25,46	26,04
Sorbs1	Sorbin and SH3 domain containing 1	25,21	26,78
Sqstm1	Sequestosome 1	22,44	23,67
Stat1	Signal transducer and activator of transcription 1	25,27	26,55
Tnf	Tumor necrosis factor	35	30,85
Tnfsf10	Tumor necrosis factor (ligand) superfamily, member 10	30,25	33,01
Txn1	Thioredoxin 1	29,83	31,49
Txnrd1	Thioredoxin reductase 1	23,64	24,75
Vegfa	Vascular endothelial growth factor A	24,44	24,81
Wisp1	WNT1 inducible signaling pathway protein 1	27,55	29,13
Wnt1	Wingless-related MMTV integration site 1	35	35
Wnt2b	Wingless related MMTV integration site 2b	30,73	30,87
Wnt3a	Wingless-related MMTV integration site 3A	35	35
Wnt5a	Wingless-related MMTV integration site 5A	25,16	26,53
Wnt6	Wingless-related MMTV integration site 6	29,58	35

Stem Cell (PAMM-405Z)

		C(t) value	
**Gene**	**Description**	**CAE**	**NEB ME**
Actb	Actin, beta	21,74	22,71
B2m	Beta-2 microglobulin	24,05	24,74
Gapdh	Glyceraldehyde-3-phosphate dehydrogenase	23,91	24,33
Gusb	Glucuronidase, beta	24,81	26,51
Hsp90ab1	Heat shock protein 90 alpha (cytosolic), class B member 1	22,58	23,02
Abcg2	ATP-binding cassette, sub-family G (WHITE), member 2	29,57	31,55
Acan	Aggrecan	35	35
Actc1	Actin, alpha, cardiac muscle 1	22,7	23,96
Adar	Adenosine deaminase, RNA-specific	30,66	30,03
Aldh1a1	Aldehyde dehydrogenase family 1, subfamily A1	21,06	22,08
Aldh2	Aldehyde dehydrogenase 2, mitochondrial	27,01	29,28
Apc	Adenomatosis polyposis coli	27,78	29,11
Ascl2	Achaete-scute complex homolog 2 (Drosophila)	35	35
Axin1	Axin 1	26,82	28,24
Bglap	Bone gamma carboxyglutamate protein	35	35
Bmp1	Bone morphogenetic protein 1	25,51	26,94
Bmp2	Bone morphogenetic protein 2	30,09	28,61
Bmp3	Bone morphogenetic protein 3	30,53	30,14
Btrc	Beta-transducin repeat containing protein	29,76	28,53
Ccna2	Cyclin A2	26,02	27,28
Ccnd1	Cyclin D1	23,92	24,82
Ccnd2	Cyclin D2	24,44	24,24
Ccne1	Cyclin E1	35	35
Cd19	CD19 antigen	32,51	34,74
Cd3d	CD3 antigen, delta polypeptide	29,02	31,52
Cd4	CD4 antigen	35	35
Cd44	CD44 antigen	25,52	26,04
Cd8a	CD8 antigen, alpha chain	32,71	35
Cd8b1	CD8 antigen, beta chain 1	32,94	35
Cdc42	Cell division cycle 42 homolog (S. cerevisiae)	24,34	25,48
Cdh1	Cadherin 1	25,09	25,32
Cdh2	Cadherin 2	32,81	32,81
Cdk1	Cyclin-dependent kinase 1	27,52	28,08
Col1a1	Collagen, type I, alpha 1	21,75	23,45
Col2a1	Collagen, type II, alpha 1	35	35
Col9a1	Collagen, type IX, alpha 1	35	35
Ctnna1	Catenin (cadherin associated protein), alpha 1	23,81	24,92
Cxcl12	Chemokine (C-X-C motif) ligand 12	25,76	28,04
Dhh	Desert hedgehog	32,45	32,99
Dll1	Delta-like 1 (Drosophila)	32,43	31,51
Dll3	Delta-like 3 (Drosophila)	34,9	29,6
Dtx1	Deltex 1 homolog (Drosophila)	31,06	31,47
Dtx2	Deltex 2 homolog (Drosophila)	26,62	29,46
Dvl1	Dishevelled, dsh homolog 1 (Drosophila)	26,46	26,61
Ep300	E1A binding protein p300	29,2	29,3
Fgf1	Fibroblast growth factor 1	25,77	26,47
Fgf2	Fibroblast growth factor 2	28,49	28,61

Fgf3	Fibroblast growth factor 3	35	35
Fgf4	Fibroblast growth factor 4	35	35
Fgfr1	Fibroblast growth factor receptor 1	24,79	26,14
Foxa2	Forkhead box A2	26,86	26,59
Frat1	Frequently rearranged in advanced T-cell lymphomas	27,22	27,8
Fzd1	Frizzled homolog 1 (Drosophila)	28,1	28,7
Gdf2	Growth differentiation factor 2	35	35
Gdf3	Growth differentiation factor 3	35	35
Gja1	Gap junction protein, alpha 1	28,56	28,67
Gjb1	Gap junction protein, beta 1	31,79	34,82
Hdac1	Histone deacetylase 1	25,3	26,53
Hdac2	Histone deacetylase 2	23,65	24,71
Hspa9	Heat shock protein 9	24,63	25,52
Igf1	Insulin-like growth factor 1	26,02	28,34
Isl1	ISL1 transcription factor, LIM/homeodomain	35	35
Jag1	Jagged 1	27,03	27,18
Kat2a	K(lysine) acetyltransferase 2A	29,63	29,63
Krt15	Keratin 15	27,47	26,7
Mme	Membrane metallo endopeptidase	28,51	30,15
Msx1	Homeobox, msh-like 1	28,89	35
Myc	Myelocytomatosis oncogene	31,99	35
Myod1	Myogenic differentiation 1	35	35
Kat8	MYST histone acetyltransferase 1	27,22	27,64
Kat7	MYST histone acetyltransferase 2	28,65	28,61
Ncam1	Neural cell adhesion molecule 1	27,49	29,19
Neurog2	Neurogenin 2	35	35
Notch1	Notch gene homolog 1 (Drosophila)	29,54	31,05
Notch2	Notch gene homolog 2 (Drosophila)	30,49	29
Numb	Numb gene homolog (Drosophila)	28,06	29,3
Pard6a	Par-6 (partitioning defective 6,) homolog alpha (C. elegans)	30,33	28,29
Pdx1	Pancreatic and duodenal homeobox 1	35	35
Ppard	Peroxisome proliferator activator receptor delta	29,49	28,81
Pparg	Peroxisome proliferator activated receptor gamma	26,75	28,86
Rb1	Retinoblastoma 1	26,11	26,77
S100b	S100 protein, beta polypeptide, neural	26,03	26,98
Sigmar1	Sigma non-opioid intracellular receptor 1	28,88	29,63
Sox1	SRY-box containing gene 1	35	35
Sox2	SRY-box containing gene 2	25,64	26,19
T	Brachyury	35	35
Tert	Telomerase reverse transcriptase	32,03	35
Tubb3	Tubulin, beta 3	35	35
Wnt1	Wingless-related MMTV integration site 1	35	35

Stem Cell Signaling (PAMM-047Z)

Gene	Description	C(t) value CAE	NEB ME
Actb	Actin, beta	21,41	23,04
B2m	Beta-2 microglobulin	22,63	24,66
Gapdh	Glyceraldehyde-3-phosphate dehydrogenase	23,06	24,65
Gusb	Glucuronidase, beta	25,81	26,63
Hsp90ab1	Heat shock protein 90 alpha (cytosolic), class B member 1	22,58	23,42
Acvr1	Activin A receptor, type 1	26,6	27,44
Acvr1b	Activin A receptor, type 1B	26,64	27,13
Acvr1c	Activin A receptor, type IC	31,75	35
Acvr2a	Activin receptor IIA	27,02	28,32
Acvr2b	Activin receptor IIB	29,91	29,86
Acvrl1	Activin A receptor, type II-like 1	26,62	29,68
Amhr2	Anti-Mullerian hormone type 2 receptor	31,69	35
Bcl9	B-cell CLL/lymphoma 9	29,35	30,49
Bcl9l	B-cell CLL/lymphoma 9-like	27,21	28,8
Bmpr1a	Bone morphogenetic protein receptor, type 1A	24,99	25,59
Bmpr1b	Bone morphogenetic protein receptor, type 1B	26,75	27,52
Bmpr2	Bone morphogenic protein receptor, type II (serine/threonine kinase)	26,62	28,04
Cdx2	Caudal type homeobox 2	35	35
Crebbp	CREB binding protein	26,51	26,86
Ctnnb1	Catenin (cadherin associated protein), beta 1	22,25	23,94
E2f5	E2F transcription factor 5	26,14	26,72
Eng	Endoglin	27,05	28,98
Ep300	E1A binding protein p300	29,23	29,87
Fgfr1	Fibroblast growth factor receptor 1	25,34	25,68
Fgfr2	Fibroblast growth factor receptor 2	24,83	25,63
Fgfr3	Fibroblast growth factor receptor 3	26,14	27,68
Fgfr4	Fibroblast growth factor receptor 4	30,82	30,7
Fzd1	Frizzled homolog 1 (Drosophila)	28,04	28,67
Fzd2	Frizzled homolog 2 (Drosophila)	28,75	31,25
Fzd3	Frizzled homolog 3 (Drosophila)	28,42	29,11
Fzd4	Frizzled homolog 4 (Drosophila)	27,68	29,54
Fzd5	Frizzled homolog 5 (Drosophila)	26,94	28,92
Fzd6	Frizzled homolog 6 (Drosophila)	28,62	29,16
Fzd7	Frizzled homolog 7 (Drosophila)	28,07	29,29
Fzd8	Frizzled homolog 8 (Drosophila)	29,57	33,69
Fzd9	Frizzled homolog 9 (Drosophila)	32,67	35
Gli1	GLI-Kruppel family member GLI1	28,59	30,87
Gli2	GLI-Kruppel family member GLI2	31,87	31,64
Gli3	GLI-Kruppel family member GLI3	35	32,9
Il6st	Interleukin 6 signal transducer	24,58	25,42
Lef1	Lymphoid enhancer binding factor 1	35	30,28
Lifr	Leukemia inhibitory factor receptor	25,78	26,14
Lrp5	Low density lipoprotein receptor-related protein 5	27,2	30,1
Lrp6	Low density lipoprotein receptor-related protein 6	25,79	27,82
Ltbp1	Latent transforming growth factor beta binding protein 1	25,71	26,74
Ltbp2	Latent transforming growth factor beta binding protein 2	27,72	28,33
Ltbp3	Latent transforming growth factor beta binding protein 3	24,88	25,85

Ltbp4	Latent transforming growth factor beta binding protein 4	26,62	29,06
Ncstn	Nicastrin	26,51	27,5
Nfat5	Nuclear factor of activated T-cells 5	26,07	26
Nfatc1	Nuclear factor of activated T-cells, cytoplasmic, calcineurin-dependent 1	26,89	28,78
Nfatc2	Nuclear factor of activated T-cells, cytoplasmic, calcineurin-dependent 2	29,67	28,32
Nfatc3	Nuclear factor of activated T-cells, cytoplasmic, calcineurin-dependent 3	26,99	27,02
Nfatc4	Nuclear factor of activated T-cells, cytoplasmic, calcineurin-dependent 4	29,07	29,78
Notch1	Notch gene homolog 1 (Drosophila)	29,04	31,52
Notch2	Notch gene homolog 2 (Drosophila)	28,75	29,15
Notch3	Notch gene homolog 3 (Drosophila)	28,55	29,08
Notch4	Notch gene homolog 4 (Drosophila)	28,9	30,17
Psen1	Presenilin 1	26,93	27,79
Psen2	Presenilin 2	28,04	29,84
Psenen	Presenilin enhancer 2 homolog (C. elegans)	22,46	23,29
Ptch1	Patched homolog 1	30,09	28,54
Ptchd2	Patched domain containing 2	35	35
Pygo2	Pygopus 2	27,61	27,32
Rbl1	Retinoblastoma-like 1 (p107)	28,21	29,67
Rbl2	Retinoblastoma-like 2	27,71	29,92
Rbpjl	Recombination signal binding protein for immunoglobulin κ J region-like	31,03	34,53
Rgma	RGM domain family, member A	27,92	29,12
Smad1	MAD homolog 1 (Drosophila)	26,95	27,17
Smad2	MAD homolog 2 (Drosophila)	25,95	25,99
Smad3	MAD homolog 3 (Drosophila)	27,69	27,61
Smad4	MAD homolog 4 (Drosophila)	23,92	26,08
Smad5	MAD homolog 5 (Drosophila)	27,28	28,05
Smad6	MAD homolog 6 (Drosophila)	27,06	28,67
Smad7	MAD homolog 7 (Drosophila)	28,79	30,71
Smad9	MAD homolog 9 (Drosophila)	32,43	30,92
Smo	Smoothened homolog (Drosophila)	30,14	31,47
Sp1	Trans-acting transcription factor 1	25,79	26,79
Stat3	Signal transducer and activator of transcription 3	25,42	26,27
Sufu	Suppressor of fused homolog (Drosophila)	28,63	29,15
Tcf7	Transcription factor 7, T-cell specific	29,72	29,44
Tcf7l1	Transcription factor 7-like 1 (T-cell specific, HMG box)	29,49	31,75
Tcf7l2	Transcription factor 7-like 2, T-cell specific, HMG-box	27,34	28,46
Tgfbr1	Transforming growth factor, beta receptor I	26,75	26,56
Tgfbr2	Transforming growth factor, beta receptor II	25,29	26,48
Tgfbr3	Transforming growth factor, beta receptor III	28,29	30,14
Tgfbrap1	Transforming growth factor, beta receptor associated protein 1	29,33	30,92
Vangl2	Vang-like 2 (van gogh, Drosophila)	30,08	35
Zeb2	Zinc finger E-box binding homeobox 2	25,84	28,03

TGFβ/BMP Pathway (PAMM-035Z)

Gene	Description	C(t) value CAE	NEB ME
Actb	Actin, beta	20,52	23,02
B2m	Beta-2 microglobulin	23,23	25,79
Gapdh	Glyceraldehyde-3-phosphate dehydrogenase	21,47	24,77
Gusb	Glucuronidase, beta	24,16	26,28
Hsp90ab1	Heat shock protein 90 alpha (cytosolic), class B member 1	21,11	23,28
Acvr1	Activin A receptor, type 1	25,54	27,56
Acvr2a	Activin receptor IIA	24,95	28,01
Acvrl1	Activin A receptor, type II-like 1	25,35	29,89
Amh	Anti-Mullerian hormone	34,57	35
Amhr2	Anti-Mullerian hormone type 2 receptor	31,7	35
Atf4	Activating transcription factor 4	25,89	27,98
Bambi	BMP and activin membrane-bound inhibitor, homolog (Xenopus laevis)	24,2	27,76
Bglap2	Bone gamma-carboxyglutamate protein 2	35	35
Bmp1	Bone morphogenetic protein 1	23,07	26,01
Bmp2	Bone morphogenetic protein 2	30,45	29,83
Bmp3	Bone morphogenetic protein 3	30,17	33,32
Bmp4	Bone morphogenetic protein 4	24,48	27,44
Bmp5	Bone morphogenetic protein 5	25,86	27,01
Bmp6	Bone morphogenetic protein 6	24,08	29,27
Bmp7	Bone morphogenetic protein 7	27,95	27
Bmper	BMP-binding endothelial regulator	26,6	33,78
Bmpr1a	Bone morphogenetic protein receptor, type 1A	23,81	25,48
Bmpr1b	Bone morphogenetic protein receptor, type 1B	27,11	26,75
Bmpr2	Bone morphogenic protein receptor, type II (serine/threonine kinase)	23,96	28,7
Cdkn1a	Cyclin-dependent kinase inhibitor 1A (P21)	23,68	26,25
Cdkn1b	Cyclin-dependent kinase inhibitor 1B	23,97	26,07
Cdkn2b	Cyclin-dependent kinase inhibitor 2B (p15, inhibits CDK4)	26,6	29,81
Chrd	Chordin	27,76	28,35
Col1a1	Collagen, type I, alpha 1	20,59	23,54
Col1a2	Collagen, type I, alpha 2	18,62	21,11
Dcn	Decorin	23	26,75
Dlx2	Distal-less homeobox 2	35	35
Emp1	Epithelial membrane protein 1	23,11	26,7
Eng	Endoglin	22,95	29,29
Fos	FBJ osteosarcoma oncogene	25,76	26,47
Fst	Follistatin	29,85	31,53
Gadd45b	Growth arrest and DNA-damage-inducible 45 beta	28,99	30,04
Gdf1	Growth differentiation factor 1	30,52	35
Gdf2	Growth differentiation factor 2	35	35
Gdf3	Growth differentiation factor 3	35	35
Gdf5	Growth differentiation factor 5	31,47	32,96
Gdf6	Growth differentiation factor 6	35	35
Gdf7	Growth differentiation factor 7	34,54	34,8
Gsc	Goosecoid homeobox	32,45	35
Herpud1	Homocysteine-, ER stress-inducible, ubiquitin-like domain member 1	28,3	30,82
Id1	Inhibitor of DNA binding 1	21,22	26,19
Id2	Inhibitor of DNA binding 2	23,46	27,5

Ifrd1	Interferon-related developmental regulator 1	22,7	24,56
Igf1	Insulin-like growth factor 1	24,71	27,58
Igfbp3	Insulin-like growth factor binding protein 3	22,91	26,89
Il6	Interleukin 6	35	35
Inha	Inhibin alpha	27,09	27,16
Inhba	Inhibin beta-A	28,31	28,79
Jun	Jun oncogene	25,88	27,45
Junb	Jun-B oncogene	31,97	32,64
Lefty1	Left right determination factor 1	27,69	32,09
Ltbp1	Latent transforming growth factor beta binding protein 1	24,32	27,07
Ltbp2	Latent transforming growth factor beta binding protein 2	24,77	28,51
Ltbp4	Latent transforming growth factor beta binding protein 4	25,55	30
Mecom	MDS1 and EVI1 complex locus	24,77	25,98
Myc	Myelocytomatosis oncogene	28,51	30,29
Nodal	Nodal	33,19	35
Nog	Noggin	35	35
Pdgfb	Platelet derived growth factor, B polypeptide	24	27,99
Plau	Plasminogen activator, urokinase	28,02	32,34
Runx1	Runt related transcription factor 1	24,83	24,91
Serpine1	Serine (or cysteine) peptidase inhibitor, clade E, member 1	26,2	28,44
Smad1	MAD homolog 1 (Drosophila)	24,79	27,44
Smad2	MAD homolog 2 (Drosophila)	23,68	25,52
Smad3	MAD homolog 3 (Drosophila)	25,1	27,46
Smad4	MAD homolog 4 (Drosophila)	24,66	26,14
Smad5	MAD homolog 5 (Drosophila)	25	27,43
Smad7	MAD homolog 7 (Drosophila)	26,57	30,69
Smurf1	SMAD specific E3 ubiquitin protein ligase 1	25,15	27,22
Sox4	SRY-box containing gene 4	30,88	35
Stat1	Signal transducer and activator of transcription 1	24,17	27,06
Tdgf1	Teratocarcinoma-derived growth factor 1	35	35
Tgfb1	Transforming growth factor, beta 1	23,06	26,67
Tgfb1i1	Transforming growth factor beta 1 induced transcript 1	22,53	25,65
Tgfb2	Transforming growth factor, beta 2	27,55	27,97
Tgfb3	Transforming growth factor, beta 3	26,17	27,47
Tgfbi	Transforming growth factor, beta induced	22,79	26,14
Tgfbr1	Transforming growth factor, beta receptor I	24,58	27,09
Tgfbr2	Transforming growth factor, beta receptor II	25,26	28,2
Tgfbr3	Transforming growth factor, beta receptor III	29,31	31,61
Tgfbrap1	Transforming growth factor, beta receptor associated protein 1	27,34	31,6
Thbs1	Thrombospondin 1	24	24,12
Tnfsf10	Tumor necrosis factor (ligand) superfamily, member 10	27,66	34,35
Tsc22d1	TSC22 domain family, member 1	20,89	22,68

TGFβ Signaling Targets (PAMM-235Z)

Gene	Description	C(t) value CAE	NEB ME
Actb	Actin, beta	15,83	24,09
B2m	Beta-2 microglobulin	19,14	26,16
Gapdh	Glyceraldehyde-3-phosphate dehydrogenase	16,65	25,22
Gusb	Glucuronidase, beta	21,49	28,18
Hsp90ab1	Heat shock protein 90 alpha (cytosolic), class B member 1	17,49	24,67
Acta2	Actin, alpha 2, smooth muscle, aorta	16,19	24,05
Acvr1	Activin A receptor, type 1	21,94	28,45
Acvrl1	Activin A receptor, type II-like 1	21,22	31,69
Agt	Angiotensinogen (serpin peptidase inhibitor, clade A, member 8)	25,33	35
Aipl1	Aryl hydrocarbon receptor-interacting protein-like 1	28,96	35
Ar	Androgen receptor	24,61	35
Atf3	Activating transcription factor 3	25,34	33,34
Atf4	Activating transcription factor 4	22,5	30,84
Bach1	BTB and CNC homology 1	27,89	35
Bcl2l1	Bcl2-like 1	20,79	27,74
Bdnf	Brain derived neurotrophic factor	24,08	35
Bhlhe40	Basic helix-loop-helix family, member e40	22,74	30,51
Brd2	Bromodomain containing 2	20,51	27,24
Cdc6	Cell division cycle 6 homolog (S. cerevisiae)	23,83	29,63
Cdkn1b	Cyclin-dependent kinase inhibitor 1B	20,5	27,61
Cebpb	CCAAT/enhancer binding protein (C/EBP), beta	21,04	27,5
Creb1	CAMP responsive element binding protein 1	21,87	28,37
Crebbp	CREB binding protein	21,69	28,61
Cryab	Crystallin, alpha B	17,95	27,71
Ctnnb1	Catenin (cadherin associated protein), beta 1	18,88	25,53
Dnaja1	DnaJ (Hsp40) homolog, subfamily A, member 1	19,52	26,07
E2f4	E2F transcription factor 4	21,33	27,93
Emp1	Epithelial membrane protein 1	17,61	28,85
Eng	Endoglin	19,63	30,53
Ep300	E1A binding protein p300	25,54	32,44
Ephb2	Eph receptor B2	23,11	27,86
Fn1	Fibronectin 1	19,15	28,9
Fos	FBJ osteosarcoma oncogene	22,15	28,86
Furin	Furin (paired basic amino acid cleaving enzyme)	21,64	30,17
Gadd45b	Growth arrest and DNA-damage-inducible 45 beta	25,34	31,7
Gli2	GLI-Kruppel family member GLI2	26,08	34,53
Gtf2i	General transcription factor II I	19,51	26,31
Herpud1	Homocysteine-, ER stress-inducible, ubiquitin-like domain member 1	24,57	33,74
Hes1	Hairy and enhancer of split 1 (Drosophila)	20,87	27,75
Hey1	Hairy/enhancer-of-split related with YRPW motif 1	20,99	28,09
Hmox1	Heme oxygenase (decycling) 1	24,59	32,42
Id1	Inhibitor of DNA binding 1	19,25	29,08
Id2	Inhibitor of DNA binding 2	20,79	29,64
Id3	Inhibitor of DNA binding 3	20,51	32,21
Ifrd1	Interferon-related developmental regulator 1	21,78	28,12
Il10	Interleukin 10	30,94	35
Klf10	Kruppel-like factor 10	24,25	33,81

Map3k7	Mitogen-activated protein kinase kinase kinase 7	20,9	27,85
Mapk14	Mitogen-activated protein kinase 14	21,06	28,08
Mapk8	Mitogen-activated protein kinase 8	21,8	29,04
Mbd1	Methyl-CpG binding domain protein 1	22,56	30,57
Mmp2	Matrix metallopeptidase 2	19,62	27,49
Msx2	Homeobox, msh-like 2	28,13	35
Myc	Myelocytomatosis oncogene	23,7	30,09
Myod1	Myogenic differentiation 1	28,46	35
Nfib	Nuclear factor I/B	21,74	29,27
Nfkbia	Nuclear factor of κ light polypeptide gene enhancer in B-cells inhibitor α	21,88	29,69
Notch1	Notch gene homolog 1 (Drosophila)	23,2	31,56
Pdgfa	Platelet derived growth factor, alpha	21,67	28,33
Plg	Plasminogen	32,06	35
Ppara	Peroxisome proliferator activated receptor alpha	22,05	32,41
Ptgs2	Prostaglandin-endoperoxide synthase 2	26,87	30,81
Pthlh	Parathyroid hormone-like peptide	26	35
Ptk2	PTK2 protein tyrosine kinase 2	20,85	27,47
Ptk2b	PTK2 protein tyrosine kinase 2 beta	22,79	29,24
Rad21	RAD21 homolog (S. pombe)	19,72	26,47
Rara	Retinoic acid receptor, alpha	22,64	28,93
Rbl1	Retinoblastoma-like 1 (p107)	22,99	29,44
Rhoa	Ras homolog gene family, member A	18,63	27,01
Rhob	Ras homolog gene family, member B	19,36	28,76
Runx1	Runt related transcription factor 1	21,59	26,68
S100a8	S100 calcium binding protein A8 (calgranulin A)	20,65	32,13
Serpine1	Serine (or cysteine) peptidase inhibitor, clade E, member 1	23,62	32,76
Shh	Sonic hedgehog	24,03	30,12
Smad1	MAD homolog 1 (Drosophila)	21,75	29,15
Smad3	MAD homolog 3 (Drosophila)	21,98	29,72
Smad5	MAD homolog 5 (Drosophila)	21,95	30,44
Smad6	MAD homolog 6 (Drosophila)	20,65	30,64
Snai1	Snail homolog 1 (Drosophila)	27,42	34,92
Sox4	SRY-box containing gene 4	24,71	34,24
Sp1	Trans-acting transcription factor 1	20,89	27,14
Srebf2	Sterol regulatory element binding factor 2	22,89	30,16
Tgfb2	Transforming growth factor, beta 2	23,22	34,7
Tgfbr2	Transforming growth factor, beta receptor II	21	28,87
Thbs1	Thrombospondin 1	21,49	26,99
Tnfsf10	Tumor necrosis factor (ligand) superfamily, member 10	23,96	33,69
Txnip	Thioredoxin interacting protein	17,75	25,68
Vegfa	Vascular endothelial growth factor A	18,99	26,79
Wfs1	Wolfram syndrome 1 homolog (human)	22,67	29,91

Wnt Signaling Pathway (PAMM-043Z)

Gene	Description	C(t) value CAE	NEB ME
Actb	Actin, beta	22,01	23,53
B2m	Beta-2 microglobulin	24,72	26,15
Gapdh	Glyceraldehyde-3-phosphate dehydrogenase	23,36	24,87
Gusb	Glucuronidase, beta	27,13	26,89
Hsp90ab1	Heat shock protein 90 alpha (cytosolic), class B member 1	22,9	23,83
Aes	Amino-terminal enhancer of split	22,24	23,66
Apc	Adenomatosis polyposis coli	28,28	30,72
Axin1	Axin 1	28,5	30,02
Axin2	Axin2	26,97	27,48
Bcl9	B-cell CLL/lymphoma 9	31,19	30,78
Btrc	Beta-transducin repeat containing protein	29,87	31,53
Ccnd1	Cyclin D1	24,98	25,09
Ccnd2	Cyclin D2	24,65	24,8
Csnk1a1	Casein kinase 1, alpha 1	24,15	25,09
Csnk2a1	Casein kinase 2, alpha 1 polypeptide	26,76	26,86
Ctbp1	C-terminal binding protein 1	25,18	26,51
Ctnnb1	Catenin (cadherin associated protein), beta 1	23,47	24,53
Ctnnbip1	Catenin beta interacting protein 1	28,19	29,96
Daam1	Dishevelled associated activator of morphogenesis 1	30,18	30,7
Dab2	Disabled homolog 2 (Drosophila)	28,59	31,73
Dixdc1	DIX domain containing 1	29,32	28,78
Dkk1	Dickkopf homolog 1 (Xenopus laevis)	35	35
Dkk3	Dickkopf homolog 3 (Xenopus laevis)	28,89	30,61
Dvl1	Dishevelled, dsh homolog 1 (Drosophila)	26,84	27,24
Dvl2	Dishevelled 2, dsh homolog (Drosophila)	29,02	32,96
Ep300	E1A binding protein p300	31,32	29,84
Fbxw11	F-box and WD-40 domain protein 11	26,68	27,58
Fbxw4	F-box and WD-40 domain protein 4	27,21	28,8
Fgf4	Fibroblast growth factor 4	35	35
Fosl1	Fos-like antigen 1	35	35
Foxn1	Forkhead box N1	35	35
Frat1	Frequently rearranged in advanced T-cell lymphomas	28,62	29,59
Frzb	Frizzled-related protein	31,34	35
Fzd1	Frizzled homolog 1 (Drosophila)	29,06	31,43
Fzd2	Frizzled homolog 2 (Drosophila)	29,06	30,21
Fzd3	Frizzled homolog 3 (Drosophila)	30,6	29,22
Fzd4	Frizzled homolog 4 (Drosophila)	29,25	30,83
Fzd5	Frizzled homolog 5 (Drosophila)	29,22	29,1
Fzd6	Frizzled homolog 6 (Drosophila)	30,33	29,76
Fzd7	Frizzled homolog 7 (Drosophila)	28,17	29,85
Fzd8	Frizzled homolog 8 (Drosophila)	32,05	35
Fzd9	Frizzled homolog 9 (Drosophila)	35	35
Gsk3b	Glycogen synthase kinase 3 beta	26,16	26,53
Jun	Jun oncogene	26,63	28,19
Kremen1	Kringle containing transmembrane protein 1	26,23	28,61
Lef1	Lymphoid enhancer binding factor 1	30,09	35
Lrp5	Low density lipoprotein receptor-related protein 5	27,67	29,15

Lrp6	Low density lipoprotein receptor-related protein 6	26,92	27,69
Mapk8	Mitogen-activated protein kinase 8	27,47	27,64
Mmp7	Matrix metallopeptidase 7	35	35
Myc	Myelocytomatosis oncogene	30,27	29,97
Nfatc1	Nuclear factor of activated T-cells, cytoplasmic, calcineurin-dependent 1	27,96	29,1
Nkd1	Naked cuticle 1 homolog (Drosophila)	26,26	27,77
Nlk	Nemo like kinase	27,94	28,64
Pitx2	Paired-like homeodomain transcription factor 2	35	35
Porcn	Porcupine homolog (Drosophila)	27,11	29,31
Ppard	Peroxisome proliferator activator receptor delta	31,53	30,04
Prickle1	Prickle homolog 1 (Drosophila)	29,25	31,22
Pygo1	Pygopus 1	28,58	31,57
Rhoa	Ras homolog gene family, member A	24,69	26,36
Rhou	Ras homolog gene family, member U	29,66	29,8
Ruvbl1	RuvB-like protein 1	25,03	26,58
Sfrp1	Secreted frizzled-related protein 1	30,86	32,45
Sfrp2	Secreted frizzled-related protein 2	28,98	28,84
Sfrp4	Secreted frizzled-related protein 4	35	34,43
Sox17	SRY-box containing gene 17	32,68	35
Tcf7	Transcription factor 7, T-cell specific	30,9	31,43
Tcf7l1	Transcription factor 7-like 1 (T-cell specific, HMG box)	30,2	33,06
Tle1	Transducin-like enhancer of split 1, homolog of Drosophila E(spl)	27,21	27,95
Vangl2	Vang-like 2 (van gogh, Drosophila)	33,04	35
Wif1	Wnt inhibitory factor 1	26,64	27,23
Wisp1	WNT1 inducible signaling pathway protein 1	29,91	29,79
Wnt1	Wingless-related MMTV integration site 1	35	35
Wnt10a	Wingless related MMTV integration site 10a	35	35
Wnt11	Wingless-related MMTV integration site 11	26,87	27,42
Wnt16	Wingless-related MMTV integration site 16	31,69	28,46
Wnt2	Wingless-related MMTV integration site 2	31,55	35
Wnt2b	Wingless related MMTV integration site 2b	31,83	35
Wnt3	Wingless-related MMTV integration site 3	35	35
Wnt3a	Wingless-related MMTV integration site 3A	32,21	35
Wnt4	Wingless-related MMTV integration site 4	29,62	32,89
Wnt5a	Wingless-related MMTV integration site 5A	27,59	29,66
Wnt5b	Wingless-related MMTV integration site 5B	30,82	31,78
Wnt6	Wingless-related MMTV integration site 6	31,8	35
Wnt7a	Wingless-related MMTV integration site 7A	35	35
Wnt7b	Wingless-related MMTV integration site 7B	32,17	31,27
Wnt8a	Wingless-related MMTV integration site 8A	35	35
Wnt8b	Wingless related MMTV integration site 8b	35	35
Wnt9a	Wingless-type MMTV integration site 9A	33,76	32,47
